# Costs and benefits of phytoplankton motility

**Published:** 2025-03-18

**Authors:** Peyman Fahimi, Andrew J. Irwin, Michael Lynch

**Affiliations:** (a) Department of Mathematics & Statistics, Dalhousie University, Halifax, NS B3H4R2 Canada.; (b) Center for Mechanisms of Evolution, Biodesign Institute, Arizona State University, Tempe, AZ 85287 USA.

**Keywords:** Phytoplankton, swimming speed, sinking speed, energetic cost, motility benefit

## Abstract

The motility skills of phytoplankton have evolved and persisted over millions of years, primarily in response to factors such as nutrient and light availability, temperature and viscosity gradients, turbulence, and predation pressure. Phytoplankton motility is broadly categorized into swimming and buoyancy regulation. Despite studies in the literature exploring the motility costs and benefits of phytoplankton, there remains a gap in our integrative understanding of direct and indirect energy expenditures, starting from when an organism initiates movement due to any biophysical motive, to when the organism encounters intracellular and environmental challenges. Here we gather all available pieces of this puzzle from literature in biology, physics, and oceanography to paint an overarching picture of our current knowledge. The characterization of sinking and rising behavior as passive motility has resulted in the concept of sinking and rising internal efficiency being overlooked. We define this efficiency based on any energy dissipation associated with processes of mass density adjustment, as exemplified in structures like frustules and vacuoles. We propose that sinking and rising are active motility processes involving non-visible mechanisms, as species demonstrate active and rapid strategies in response to turbulence, predation risk, and gradients of nutrients, light, temperature, and viscosity. Identifying intracellular buoyancy-regulating dissipative processes offers deeper insight into the motility costs relative to the organism’s total metabolic rate.

## 1. Introduction

Phytoplankton, organisms capable of photosynthesis and mixotrophy, play pivotal roles in carbon cycling and primary production within the ocean ecosystem [1]. Investigating their locomotion—both swimming and sinking/rising—provides insights into marine and freshwater ecology, including biogeochemical cycling [2], dynamics of food webs [3], biological carbon pumps [4], population growth rates [5], and phenomena like marine snow and blooming [6].

In this review, we delve into the costs and benefits associated with the locomotion of phytoplankton species. Specifically, we explore their swimming and buoyancy regulation over short distances in patchy aquatic environments and over long distances as part of migratory behaviors. Our examination encompasses both biological and physical dimensions of this phenomenon of oceanography shedding light on the gaps and potential future research directions.

There are notable differences between swimming and sinking/rising, resulting in varying cost-benefit analyses across species. For example, diatoms demonstrate sinking and rising behaviors, whereas dinoflagellates possess the capability for both swimming and buoyancy regulation. This differentiation might address certain unresolved issues in the literature concerning variations in population growth rates among different phyla. For example, why do dinoflagellates typically exhibit lower population growth rates compared to diatoms of similar size, despite having higher protein, chlorophyll, and carbohydrate content [7,8]? The deeper question is why an organism would want to do this, as selection puts a primacy on offspring production. Presumably, what is lost in growth rate is compensated by higher survival, i.e., avoidance of predators? Below, we list a series of physiological disadvantages that may increase metabolic costs and force the organism to allocate less energy to growth.

Dinoflagellates possess a high DNA content, lower energy-to-mass conversion efficiency, and a low chlorophyll-to-carbon (Chl:C) ratio [8]. They rely on energetically costly defense mechanisms. For instance, under predation risk, some dinoflagellates can produce bioluminescence as a protective measure, which requires approximately 60 ATP molecules per photon [9,10]. Each bioluminescent flash can last 0.1 to 0.5 s and can involve the production of 10⁷ to 10^11^ photons per second [11], equating to a minimum of 60 × 0.1 × 10^7^ = 6 × 10^7^ to a maximum of 60 × 0.5 × 10^11^ = 3 × 10^12^ ATP molecules per flash. Another defense mechanism is toxin production, such as saxitoxin, which requires at least 132 moles of ATP to synthesize one mole of the substance [12]. The number of moles of saxitoxin per cell can be determined based on its nitrogen content. Assuming 10^−11^ g N per cell [12], and given the molecular formula of saxitoxin (C₁₀H₁₇N₇O₄), where the nitrogen content per mole of saxitoxin is 7 × 14 g, we calculate 10^−11^ / (7 × 14) = 1.02 × 10^−13^ mol saxitoxin per cell. Multiplying by 132 moles of ATP per mole of saxitoxin gives 1.3 × 10^−11^ mol ATP per cell, which, when converted using Avogadro’s number, results in 8.1 × 10^12^ ATP molecules per cell. Under nutrient-limited conditions, this significantly reduces the cell-division rate by more than 20% [12]. Additionally, the respiration-to-photosynthetic rate ratio in dinoflagellates is, on average, higher than that of diatoms [13]. This indicates that dinoflagellates produce fewer ATP molecules in their chloroplasts relative to diatoms, potentially fixing less carbon for biomass production. Consequently, this lower efficiency contributes to their reduced growth rates [14].

It has been suggested that dinoflagellates coexist with other phytoplankton species by using their swimming abilities to counteract physiological disadvantages [15]. For instance, an organism under nutrient limitation can temporarily rely on internal quota resources, but once these are depleted, its growth rate declines [16], whereas the swimming abilities of mixotrophic dinoflagellates allow them to maintain a positive net growth rate even under extremely low nutrient availability [17]. However, does the benefit of swimming always outweigh its costs? In this study, through an extensive analysis of swimming costs, we demonstrate that these costs can be remarkably high relative to the total metabolic rate of the organism, even under conditions favorable for active growth. The significant contribution of such high metabolic costs may compel the organism to allocate less energy to growth, resulting in relatively lower population growth rates.

The cost of motility in phytoplankton is initially influenced by structural differences among species, including both intracellular and cell surface structures. These costs consist of construction and operational expenses. Operational costs can be particularly high due to dissipative processes. Dissipation refers to the process by which energy is gradually spread throughout a system or lost, commonly in the form of heat, or converted into forms that are less efficient for performing work. The dissipative costs determine motility efficiency, which can be divided into external and internal efficiency. Motility external efficiency is influenced by cell surface structures, including the geometric shape of the cell body and the external features of motility-related structures, such as flagella (e.g., their length, number, geometry, angle, and arrangement). In contrast, motility internal efficiency is governed by dissipative processes within intracellular structures, such as the axoneme, molecular motors, type IV pili, frustule, vacuole, gas vesicle, and coccolith.

The impact of species’ geometric shape on their motility external efficiency differs between swimmers and sinkers. Observations suggest that among swimmers, prolate-shaped microorganisms exhibit higher swimming external efficiencies compared to compact or oblate-shaped ones [18]. Conversely, experimental data indicate that among sinkers, spherical-shaped microorganisms predominantly demonstrate superior sinking external efficiency, resulting in higher sinking rates compared to species with other geometric shapes [19]. To the best of our knowledge, there is no solid foundational theory explaining this discrepancy between swimmers and sinkers. Besides the geometric shape of their bodies, the motility external efficiency of phytoplankton swimmers is influenced by factors such as the arrangement and number of active flagella, as well as their length, beat frequency, and beat amplitude. These factors determine the forces and torques generated, which propel the organism [20].

Because swimming and buoyancy regulation each arise from distinct intracellular mechanisms, the associated costs of these mechanisms also differ. Swimmers, such as flagellated phytoplankton, invest in constructing and operating flagella [21], maintaining swimming internal efficiency through flagellar bending [22], and the associated dissipation of energy. Conversely, in sinkers like diatoms, the differences in mass density between cellular components, such as vacuoles, other organelles, the cytoplasm, and structural compartments like the frustule, play significant roles in buoyancy regulation. This regulation is achieved through processes like substituting heavy ions with lighter ones and vice versa within vacuoles or modifying the composition and structure of the frustule [23,24]. Because buoyancy regulating species are frequently likened to passive particles in the literature, the assessment of energy dissipation linked to intracellular machineries has been neglected. Our perspective is that sinking and rising are active mechanisms for motility, using non-visible machinery. In this paper, we discuss the biological mechanisms involved in sinking internal efficiency, while the underlying physics needs to be formulated in future studies. The emphasis on non-visible motility mechanisms stems from the fact that determining the structure and function of visible motility machinery (such as flagella) is relatively easier with video microscopy, making it more accessible for researchers to investigate its efficiency. In contrast, studying non-visible motility mechanisms, such as those mediated by buoyancy-regulating systems like the frustule, vacuole, gas vesicle, and coccolith—found in diatoms, cyanobacteria, coccolithophores, and others—is more challenging.

The cost of motility also depends on environmental factors, movement path or trajectory, and events occurring during migration; these factors include gradients of nutrients [25], light [25], temperature [25–27], and viscosity [28], a potentially chaotic path [29], and events such as turbulence [30], predation [31], and virus risk [32].

Both slow and fast sinkers and swimmers exhibit vertical migratory behaviors [33]. Slow movers engage in continuous migration over days and week, while faster ones undergo diel migration [33]. Being fast or slow is a relative concept, enabling species to migrate between a specific depth and the ocean surface, or vice versa, within a defined timeframe, such as within a single day. During vertical migration, species face turbulent flows of water. In response, sinkers and swimmers may employ different tactics such as rapid bursts of movement [34], chain formation [35], morphology adjustments [30], or changes in orientation [30].

In regions with high viscosity gradients, the costs and benefits of hydrodynamic interactions may vary for swimmers and sinkers. In such environments, species may encounter elevated local microviscosities resulting from the combined biological activities of numerous other species, such as those found within marine aggregates, associated with extracellular polymeric substances and lysis [28], webs of colloids, and mucus sheets [36,37]. These conditions lead to the formation of heterogeneous viscosity gradients, which in turn affect the local distribution of species and the availability of nutrients [28]. The viscosity gradients arising from hydrodynamic interactions, together with electrostatic interactions from larger particles and species, can potentially trap smaller particles and species. This creates a hitchhiking-like effect, where the smaller particles or species are carried along with the larger ones, influencing their distribution and behavior within the environment.

Typically, organisms are expected to slow down in higher viscosities, potentially leading to their accumulation in a region [28]. However, reports indicate that *Chlamydomonas reinhardtii* employs tactics to reorient itself along paths of lower viscosity gradients [38], analogous to Snell’s law of refraction [39], thereby minimizing viscous dissipation. Is this strategy specific to swimmers, or do sinking species have their own strategies when facing high viscosity gradients? This is important to understand because it can influence the distribution of both swimmers and buoyancy-regulating microorganisms at different layers or depths within the ocean or lake community.

## 2. Modelling the Relationship Between Applied Force and Achieved Speed for Swimming Phytoplankton

In this section, we explore Stokes’ law, along with time-averaged point-force models from the literature, to determine the applied forces by the organism’s body and flagella, and the resulting velocity fields of various phytoplankton swimmers, including Chrysophytes, Raphidophytes, Haptophytes, Chlorophytes, and Dinoflagellates. The explicit analytical form of force-velocity relation is more accessible for flagella-mediated phytoplankton swimmers compared to those relying on internal machinery, such as cyanobacteria, including *Synechococcus* and *Synechocystis*. Therefore, for cyanobacteria, we do not provide explicit force models but rather discuss the flow of ideas and the significant results.

Phytoplankton swimmers, such as dinoflagellates, inhabit low Reynolds number regimes of fluid flow, where the dimensionless ratio between the inertial forces exerted by the swimmer and the viscous forces of the environment is significantly less than one $\left(\frac{rv\rho }{\eta }\ll 1\right)$. In this regime, viscous forces predominate, and inertia plays no substantial role in swimming. Here, r,v,ρ denote the characteristic radius, speed, and mass density of the microswimmer, while η represents the viscosity of the medium.

The Navier-Stokes equation, which governs motility in a viscous fluid, describes the momentum balance in a Newtonian fluid based on Newton’s second law. For Newtonian fluids, the viscosity remains constant, meaning the fluid’s resistance to flow does not change with the speed at which it flows or the amount of force applied. In the low Reynolds number regime, the inertial terms can be disregarded. Under these conditions, the drag force, F, acting on a spherical, rigid particle in laminar and uniform flow conditions, assuming non-interfering particles, is given by:

(1) F=6πηrv

where π≅3.14 is a constant. The power required for swimming of a spherically-shaped species is given by, $P=Fv=6\pi \eta rv^{2}$. This equation has found extensive use in the literature for estimating the swimming cost of microswimmers [26,40–42]. For a thorough derivation, readers are directed to fluid mechanics textbooks such as [43], specifically the section titled ‘Creeping Flow around a Sphere.’ For detailed, biologically relevant discussions, see ‘Foundations 16.2’ in Ref. [44].

The speed of flagellated phytoplankton and the flow fields they generate are influenced by the number of active flagella [21], flagellum length [21], beat frequency, beat amplitude, beat synchronization [45], and flagellar arrangement [20]. The data on the number and length of flagella in over 200 species of unicellular eukaryotes, including phytoplankton, and bacteria are readily accessible in [21]. As an example of the flagellar arrangement in phytoplankton species, dinoflagellates possess two flagella—one transversal and the other longitudinal (known as the trailing flagellum). Fenchel [46] observed that the trailing flagellum contributes to both the cell’s translational velocity and its rotation around an axis that is perpendicular to its length. Meanwhile, the transversal flagellum mainly induces rotation around the cell’s longitudinal axis.

Predicted swimming velocity for various phytoplankton swimmers, modeled according to the forces and torques associated with diverse flagellar arrangements [20,47] can be computed using idealized force models (Table 1). These point-force models include the stokeslet [48], puller stresslet [48,49], and three-stokeslet quadrupole [50]. They are defined based on the action of one or more time-averaged point forces applied at a reference point, with the velocity fields derived from Green’s function solutions [51]. As an example, the translational velocity of biflagellated haptophytes (last row, Table 1) incorporates two time-averaged equal point-forces applied perpendicularly to the movement direction, along with two reaction forces induced by the flow of fluid where θ represents the angular force position, R denotes the radial force position, and r is the equivalent spherical radius of the cell [47].

There are alternative forms of swimming, other than employing flagella in phytoplankton, which are not easily observable. In *Synechococcus*, locomotion is facilitated by traveling waves along the surface of the cell body [53]. These waves result from cyclic surface deformations driven by protein motors modeled as a rotating helix. Motors move faster than waves traveling on the body surface, and the waves move faster than the organism’s swimming speed. The force required to propel the organism originates from the double integration of the stress tensor, which is influenced by the fluid velocity fields across the cell membrane surface [53]. Consider a cell with a radius r of 1μm, a wave amplitude ϵr of 0.02 μm associated with waves on its surface, and a dimensionless wave number $k=\frac{2\pi r}{\lambda }$ , where λ represents the wavelength. To achieve an organism velocity v of 15 μm/s, a wave speed $c=\frac{\omega r}{k}$ of 456 μm/s is necessary, where ω represents the angular frequency, as determined by the formula $v=82.2\epsilon^{2}c$ derived in [53]. The fixed dimensionless coefficient 82.2 in this equation arises from an approximate representation of cell-surface deformations as Legendre polynomials and a fixed wave number k=16. For relatively large wave numbers, the result will change. For a complete discussion, see [53] and exploration of other models investigating the swimming velocity driven by surface-distorted waves, see [53–55]. The organism’s speed contributes to the Stokes hydrodynamic swimming cost, while the wave speed on the surface of the cell body and the speed of motor proteins contribute to energy dissipation in the internal machinery.

The freshwater cyanobacterium, *Synechocystis sp.*, exhibits a gliding or twitching-type motility with an average speed ranging from 0.09 to 0.15 μm/s [56]. This movement in water is facilitated by type IV pili (T4P), which are long, hair-like dynamic filaments on the organism’s surface. The molecular structure of T4P is detailed in [57]. Although the physics governing the velocity field patterns, thrust forces, and swimming efficiency of this cyanobacterium remain unexplored, it is suggested that its motility resembles that of soil bacteria such as *Myxococcus xanthus* [58]. The motility of *Myxococcus xanthus*, viewed as an elastically flexible cell, is modeled by several forces: the forces generated by circular nodes (motor proteins) interconnected by angular springs, viscous forces, and interaction forces between nodes and substrates [59]. The retraction-elongation velocity of a single pilus fiber is approximately 1 μm/s with an average stall force of 110 pN [60], and this velocity is contingent upon the concentration of retraction motors [61]. The stall force is the force at which the pilus fiber stops moving despite the continued application of force.

### 3. Essential Principles of Phytoplankton Movement: Sinking and Rising Dynamics

In this section, we discuss the Stokes-driven buoyancy equation, focusing on the parameters it captures completely, partially, or not at all. We explore the developed form of this equation, emphasizing the differences in mass density among various organelles of the species and their respective volumes, which can deviate from the mean mass density of the whole organism.

When a spherical rigid object falls through a fluid, three forces act upon it: 1) Gravitational Force: This force pulls the object downward and is determined by the object’s mass, $F_{\text{gravity}}=mg$, where m is the object’s mass and g is the gravitational acceleration (standard value g=9.80665 m/s^2^). For a sphere, the mass is given by $m=\rho_{c}V$, where $\rho_{c}$ is the mass density of the sphere and $V=\frac{4}{3}\pi r^{3}$ is its volume. Substituting m, we get $F_{\text{gravity}}=\frac{4}{3}\pi r^{3}\rho_{c}g.2$) Buoyant Force: This upward force arises due to the displaced fluid and is expressed as $F_{\text{buoyant}}=\rho_{w}Vg$, where $\rho_{w}$ is the mass density of the fluid. 3) Drag Force: This resistive force opposes the object’s motion through the fluid. According to Stokes’ law (applicable in the low Reynolds number regime – Equation (1)), $F_{drag}=6\pi \eta rv$, where η is the dynamic viscosity of the fluid, r is the radius of the sphere, and v is its velocity. By balancing these forces, $F_{\text{gravity}}-F_{\text{buoyant}}=F_{\text{drag}}$. Substituting the expressions for each force and solving for v, the sinking/rising velocity is:

(2) $$v=\frac{2gr^{2}\left(\rho_{c}-\rho_{w}\right)}{9\eta }$$

where $\rho_{c}-\rho_{w}$ represents the difference between the mass density of the object (e.g., a sinking or rising phytoplankton) and the fluid (e.g., seawater or freshwater). This equation assumes that phytoplankton sinkers behave similarly to inert particles; however, these living organisms possess the ability to dynamically alter their mass density, morphology, and orientation. While this equation is frequently used in the literature, it fails to encompass all the nuances of sinking species.

In general, intracellular macromolecules like proteins and carbohydrates typically possess a higher mass density (~ 1300 and 1500 kg.m^−3^ respectively) than seawater (~ 1030 kg.m^−3^), whereas lipids have a lower mass density (~ 860 kg.m^−3^) in comparison to water [62]. Phytoplankton exhibit varying density differentials relative to their environment. This density differential causes vertical migration, both upward and downward, over the course of a day, several days, or even up to a week [63]. Phytoplankton employ several mechanisms to adjust their internal density. Eukaryotic phytoplankton may replace lighter ions with heavier ones, or vice versa [23], accumulate ballast materials [23], or incorporate ammonium derivatives such as trimethyl ammonium (~ 993 kg.m^−3^) [62] into their vacuoles to reduce mass density and achieve positive buoyancy. In contrast, cyanobacteria manipulate gas vesicles [23] to regulate buoyancy. The mass density of phytoplankton typically ranges from 920 to 1085 kg/m^3^ for cyanobacteria, 1020 to 1140 kg/m^3^ for Chlorophyta, and 1009 to 1263 kg/m^3^ for Bacillariophyta [64–66].

Density variations in diatoms arise also from a complex interplay among factors such as the heavy siliceous frustule (denser than 2000 kg.m^−3^), cell shape, nutrient flux, and silica requirements, which are influenced by environmental conditions and grazing pressure [67]. The silicification process, which defines the frustule, significantly influences vertical migration of diatoms when high energy resources are not available [68,69]. When faced with nutrient limitations and iron deficiencies, diatoms tend to exhibit higher Si:C ratios, promoting faster sinking rates to access nutrient-rich deep ocean layers [67].

Although lipids have a lower mass density than water, they are not thought to play a major role in buoyancy regulation [62]. This is primarily because vacuoles [62,70], which occupy a considerable portion of the cell volume (typically 30% in relatively small diatoms to 90% in relatively large diatoms [71]), and silica-rich heavy frustules play the major role in buoyancy regulation. However, it has been reported that there is a strong positive correlation between the increased sinking speed of *Phaeodactylum tricornutum* diatoms—nearly threefold—and the rise in their lipid content under phosphate limitation or depletion [72]. This may suggest that changes in lipid composition lead to structural alterations, such as modifications in membrane rigidity, which result in changes in cell shape and size [73,74], and/or changes in the interactions between lipid droplets and vacuoles [75,76]. All of these factors could indirectly influence sinking behavior.

Large diatoms like *Nitzschia palea*, lacking swimming capabilities, can sink rapidly, reaching speeds of up to 43 m/d or 500 μm/s [77]. These speeds are variable, exhibiting an unsteady sinking pattern observed in large species like *Coscinodiscus wailesii* [34,78]. This behavior involves bursts of rapid sinking with a 1.5% change in mass density, coupled with periods of slow or near-zero speeds. Such fluctuating behavior among diatoms over timescales of seconds seems to confer a competitive advantage in nutrient assimilation [34]. Nevertheless, this fluctuating behavior appears absent under dark conditions, at least in the case of *Coscinodiscus wailesii* [79].

Theoretical models have been developed to predict the maximum sinking rate of cylindrical and spherical diatoms with greater precision than conventional Stokes law [24]. Based on the model in [24] the sinking speed of spherical diatoms is given by:

(3) $$v=\frac{2g}{9\eta }\left[\rho_{\text{cyt}}\frac{{\left(r-t\right)}^{3}}{r}+\rho_{\text{fr}}\frac{\left(3r^{2}t-3rt^{2}+t^{3}\right)}{r}-\rho_{\text{w}}r^{2}\right]$$

where $\rho_{\text{cyt}}$, $\rho_{\text{fr}}$, and $\rho_{\text{w}}$ denote the mass densities of the cytoplasm, frustule, and seawater, respectively, and $t=b_{1}r^{a_{1}}$ represents the radius-dependent frustule thickness (with dimensions of length). Here, $a_{1}$ and $b_{1}$ are scaling coefficients, and each term in the brackets has the dimension of mass per unit length (ML^−1^). The cell density is $\rho_{\text{cell}}=\rho_{\text{cyt}}\frac{V_{\text{cyt}}}{V_{\text{cell}}}+\rho_{\text{fr}}\frac{V_{\text{fr}}}{V_{\text{cell}}}$, where $V_{\text{cyt}}$, $V_{\text{fr}}$, and $V_{\text{cell}}$ represent the volumes of the cytoplasm, the frustule, and the cell. One suggestion for future research is to incorporate the significant influence of vacuole mass density ($\rho_{\text{vac}}=1017.6$ kg.m^−3^ [80]), due to their considerable volume and role in positive buoyancy [23,71,81], into Equation (3). This factor reduces overall cell density, thereby facilitating positive buoyancy [82]. The approach to do this is to add a new term in the form of $\rho_{\text{vac}}\frac{V_{\text{vac}}}{V_{\text{cell}}}$ into Equation (3). To determine the volume of the vacuole relative to the cell volume, data provided in [71] for diatoms can be used. By fitting a function to this data, the ratio $\frac{V_{\text{vac}}}{V_{\text{tot}}}$ for different cell volumes can be obtained.

One important question is: how much variation in density is possible, and how long does it take for the buoyant cell to achieve this? According to recent findings [83], the *Pyrocystis noctiluca,* a non-flagellated dinoflagellate, experiences a fast six-fold increase in cellular volume within a span of less than 10 minutes. This process involves the calcium-activated influx of *fresh* water through aquaporin channels, followed by transport to the vacuole, resulting in the reversal of gravitational sedimentation [83]. This creates a situation where the cell mass density, $\rho_{\text{cell}}=\rho_{\text{cyt}}\frac{V_{\text{cyt}}}{V_{\text{cell}}}+\rho_{\text{vac}}\frac{V_{\text{vac}}}{V_{\text{cell}}}$, becomes less than the mass density of seawater.

The sinking and rising dynamics expressed in the form of Equations (2) and (3) do not incorporate the shape factor as a parameter. In diatoms, non-spherical geometric shapes generally sink at a slower rate compared to equivalent spheres [19,84]. However, tear-drop-shaped phytoplankton serve as an exception, sinking faster than their spherical counterparts [19,84]. Apart from shape, size, and mass density, the key factor influencing sinking rate is the species’ angle of orientation. The angle of orientation depends at least on the cell’s geometric shape and intracellular mass distribution. An uneven mass distribution shifts the positions of the center of mass and the center of buoyancy, creating a torque that affects the cell’s orientation. Sinking rate is typically assessed with random orientation, leaving scant information on how sinkers like diatoms modify their morphology or orient themselves [85] for optimal sinking speed.

Theoretical models, such as the one developed by Jeffery [86], propose that rigid spheroids rotate under shear flows. The period of rotation depends on the shear rate and the shape of the rigid body. Spheres have the shortest rotation periods (rotating faster), while both prolate and oblate spheroids have longer rotation periods compared to a perfect sphere. However, experiments with chain-forming diatoms have shown that this phenomenon is more complex due to various physiological and environmental factors, including nutrient flux and the mechanical properties of the chain [87]. For instance, in *Skeletonema costatum* chains, the period of rotation increases with the length-to-width ratio, whereas no correlation between the rotation period and axis ratio is observed in *Thalassiosira nordenskioldii* [87]. In laboratory experiments [88], it has been reported that a mixture of randomly oriented pennate diatom cells with elongated shapes initially reorient themselves in the direction of gravitational acceleration in the absence of external currents. Following this, they segregate from dead or differently-sized cells and subsequently pair with healthy partners for sexual reproduction [88]. The physical mechanisms behind cell pairing in diatoms may include collective hydrodynamic interactions [88], cell surface charge [89], and electrostatic interactions [89]. Hydrodynamic instability suggests that the time required for cells to cluster is influenced by factors such as sinking speed, cell density (which can be high during blooming periods), and the characteristic size of the cells [88]. The cost associated with cell rotation follows the Stokes viscous dissipation formula, but applies to rotational motion, involving angular velocity rather than linear velocity [90].

Phytoplankton may be infected by viruses, which can alter their sinking speed. The sinking speed of infected phytoplankton, e.g., in *H. akashiwo*, may increase as a defense mechanism to remove viruses attached to their surface and may also increase due to a rise in the mass density of the host cell [91,92], or the formation of larger cell aggregates [93], thereby enhancing the rate of particulate organic matter sedimentation. Thus, viral infection not only raises the cost associated with defense mechanisms but also increases viscous dissipation due to the increased sinking speed.

Phytoplankton aggregates, such as marine snow, consist of attached clusters of live and dead cells, along with various organic and inorganic materials like detritus and minerals. These aggregates range in size from micrometers to millimeters and exhibit a fractal nature. A key factor influencing the shape, mass density, and consequently the sinking velocity of these aggregates is porosity. Porosity is described as the ratio of the volume occupied by voids, which may contain water or other fluids, to the total volume of the aggregate. In a recent study [94], the authors developed four different models to investigate the sinking velocity patterns of phytoplankton aggregates in relation to their size, considering both constant aggregate density and aggregate density based on fractal geometry. The results show that the sinking velocity patterns depend on the aggregate composition. The sinking speed of particles and aggregates increases with ocean depth, likely due to alterations in porosity and density resulting from compositional changes [95,96]. Phytoplankton in aggregates benefit from better access to nutrients and protection from speed-dependent viscous dissipation because they are moved by the aggregate rather than sinking on their own. However, they also face the drawback of higher local viscosity, which can impede their movement [28].

## 4. Phytoplankton Migration: Survival Strategies

In this section, we discuss the time-scale and length-scale of vertical migration for both relatively slow and fast phytoplankton, their role in net primary production, and the equations that determine their vertical velocity in relation to depth-dependent profiles of light and nutrient concentrations and maximum photosynthetic rate. We examine the velocity at which migrating phytoplankton reach their target depth, the impact of increasing speed on the accumulation of species at specific depths, and the influence of turbulence on diversity. Additionally, we explore the strategies phytoplankton employ in turbulent waters, such as chain formation, division into sub-populations, and changes in orientation, direction, and shape.

Phytoplankton primarily engage in migration through chemotaxis, phototaxis, and gravitaxis, which are movements in response to chemical gradients, light, and gravity, respectively. When species migrate either towards the ocean’s surface, where light intensity is higher, or to deeper depths with greater nutrient concentrations, there exists the potential for an increase in their rates of photosynthesis or nutrient uptake [40,97]. The light-rich and nutrient-rich regions are typically separated by distances of 30–120 m, and relatively fast phytoplankton species are capable of vertical diel migration within these regions [40,97].

Motility skills enable phytoplankton species to engage in diel vertical migration, observed and calculated to occur vertically up to tens of meters (5–75 m) over a 12-hour period [98]. During vertical migration, the species may also exhibit limited horizontal movement, relative to vertical migration, through diffusion alone, typically spanning up to 10 centimeters, as observed in ideal lab experiments in the absence of water currents [98]. This horizontal displacement is relatively insignificant compared to their predominant vertical movement; however, tidal currents can significantly influence the horizontal movement of species [99].

Contrary to the common assumption that most phytoplankton species lack migratory behaviors due to their relatively slow speed, Wirtz and Smith [33,63] reported that the observed depth-dependent profile of chlorophyll-a concentration [25] in the oligotrophic ocean is attributed to long-term (days to week) slow vertical migration via sinking/rising or swimming processes. This slow form of active mobility, which is considerably more challenging to track compared to diel migration, contributes to 7–28% of the ocean’s net primary production [33,63]. The variation in the nutrient-to-carbon ratio at identical ocean depths is suggested to result from distinct migration histories, involving movement either from the upper surface ocean to deeper depths or vice versa [33].

Both light intensity and nutrient concentration vary with ocean depth during migration. The ratio between the light intensity at the lower station and that at the upper station, $\frac{I}{I_{0}}$, is determined by the following equation [40,63] in which $k_{I}$ is the light attenuation coefficient and Δz is the migration distance:

(4) $$\frac{I}{I_{0}}={\text{e}}^{-k_{I}\text{Δ}z}$$

The opposite relationship applies to the nutrient uptake rate ratio, $\frac{V_{N}}{V_{N_{0}}}$, in the following manner:

(5) $$\frac{V_{N}}{V_{N_{0}}}={\text{e}}^{k_{N}\text{Δ}z}$$

The nutrient gradient results from the separation between the mixed layer and the chemocline in the upper ocean. While the depth of the chemocline can vary depending on the ecosystem, it is generally assumed to occur at depths ranging from 60 to 180 meters. The nitrate concentration data reveals that the nutrient attenuation coefficient is three times greater than the light attenuation coefficient, $k_{N}=3k_{I}$, leading to $\frac{V_{N}}{V_{N_{0}}}={\left(\frac{I}{I_{0}}\right)}^{-3}$ [63]. The temporal evolution of migration distance gives rise to vertical velocity, where a positive or negative sign represents upward or downward movement, respectively. As the organism’s swimming or sinking speed increases, the metabolic cost of motility rises, potentially reducing the population growth rate. Additionally, when an organism moves relatively faster, it may have less time to absorb light for photosynthesis when descending and less time to uptake nutrients when ascending. Considering these factors, the organism must balance its energy allocation between motility and ensuring adequate exposure to light and nutrients at the target depth. This balance can be described using a physiological performance function that relates motility speed, growth rate, and maximum photosynthesis rate within a quota-balance model [63]. This method assists in identifying the average growth rate, taking into account both migration history and environmental conditions.

Klausmeier and Litchman [100] developed a dynamic mass-balance model that considers swimming/sinking speed as a parameter, integrating factors such as growth rate under light and nutrient limitation, loss rate, eddy diffusion, and active movement to predict the vertical distribution of phytoplankton in poorly mixed water columns. Active movement is expressed as a term in a time-dependent biomass-balance equation, represented as a one-dimensional gradient of the product of speed, biomass (B), and the gradient of the growth rate, written as $\frac{\partial }{\partial z}\left(v\frac{\partial \mu }{\partial z}B\right)$. Their findings demonstrate that as phytoplankton speed increases, they tend to concentrate within a narrow layer at a specific depth, where they experience an equal limitation of light and nutrients. This depth represents an evolutionarily stable strategy [100].

Turbulence is an inherent characteristic of oceanic waters that significantly affects phytoplankton, impacting their mortality rate, motility costs, distribution, and diversity [101]. As a result, phytoplankton must develop strategies to adapt to turbulent conditions. In typical estimations, it’s often assumed that the speed of both vertical and horizontal water currents is insignificant compared to that of microswimmers/sinkers, simplifying calculations. However, when confronted with relatively faster water currents, such as rip currents, eddies, and turbulence, microswimmers and sinkers employ strategies that have been observed in lab experiments. The fundamental physics governing some idealized strategies of a model swimmer, such as swimming towards safer points, minimizing swimming time and path in parallel currents, source currents, and sink currents, is explored in [102].

Migrating phytoplankton demonstrate the ability to re-orient their swimming trajectory in response to turbulence [103] or engage in collective behavior by forming elongated chains, as observed in diatoms, cyanobacteria, and dinoflagellates within the water column [103–105]. The formation of chains enhances swimming/sinking speeds particularly in weak to moderately turbulent water flows [105]. This occurs because as the number of cells in a chain increases, the collective propulsive force outweighs the hydrodynamic drag acting on the chain [35]. The swimming speed of the chain can be determined by considering both the number of cells comprising the chain and the chain’s shape factor [105] as following:

(6) $$v_{\text{c}}^{\left(n\right)}=n^{\left(\frac{2}{3}\right)}\frac{v_{\text{c}}^{\left(1\right)}}{K}$$

where $v_{\text{c}}^{\left(n\right)}$, n, $v_{\text{c}}^{\left(1\right)}$, and K represent, respectively, the chain swimming speed, the count of rigidly attached spherical cells, the swimming speed of a single species, and the shape correction factor.

In turbulent hydrodynamic conditions, phytoplankton, such as dinoflagellates and raphidophytes, may divide into two subpopulations within approximately 30 minutes [30]. One group propels upwards while the other navigates downwards, facilitated by geometric shape adjustments to their motility mechanisms [30]. To model the dynamic morphological changes, particularly the fore-aft asymmetry discussed in [30], it is necessary to investigate the center of buoyancy, center of mass, center of hydrodynamic stress, and the organism’s orientational stability. While such rapid responses to severe environmental shifts may expose the microorganism to elevated cellular stress—characterized by nitric oxide (NO) production—and incur significant costs, they underscore the species’ advanced survival strategies.

## 5. Trade-offs of Phytoplankton Movement: A Cost-Benefit Perspective

Swimming and buoyancy regulation have metabolic costs for both the capacity (e.g. biosynthesis of flagella) and operation. We can analyze our understanding of the factors influencing migratory behavior by analyzing these costs and comparing them to the benefits of motility.

### 5.1. Benefit Analysis

Nutrient uptake rate experiences an enhancement during swimming or sinking/rising due to advection, as determined by the dimensionless Sherwood number [106,107]:

(7) $$Sh=\frac{Q}{4\pi rDC_{\infty }}$$

in which Q, r, D, and $C_{\infty }$ represent, respectively, the nutrient flux, cell radius, diffusion coefficient, and nutrient concentration at a distance far from the cell. A stationary cell is characterized by an Sh of 1 while an Sh of 1.2 signifies a 20% enhancement in nutrient uptake rate attributed to swimming/sinking. For example, empirical investigations of the flow field around the dinoflagellate *Dinophysis acuta* (79 μm in length, 54 μm in width, 104 μm/s swimming speed, 𝐷 = 10^−9^ m²/s, with constant nutrient concentration far from the cell and zero nutrient concentration at the cell surface) show that swimming contributes to a substantial 75% increase in nutrient uptake rate compared to diffusion alone [107].

To calculate the Sherwood number, one can employ the correlation between the Sherwood number and the Peclet number, $\text{Pe}=\frac{rv}{D}$, developed for a spherical steady swimmer [108], a monoflagellated organism [106], and a biflagellated organism [109]. An example of such an empirical relationship for a sinking sphere found in the literature is [110]:

(8) $$\text{Sh}=\frac{1}{2}\left(1+{\left(1+2Pe\right)}^{1/3}\right)$$

Given that the speed and characteristic radius of phytoplankton species are documented in the literature, it is straightforward to estimate the Sherwood number and consequently evaluate the enhancement in nutrient uptake attributed to swimming or sinking using Equation (8). For example, for the spherical-shaped sinking coccolithophore *Calcidiscus leptoporus* with a radius of approximately 10 μm and a mean sinking rate of 4.3 m/d [77], the Peclet number is calculated as: $\text{Pe}=\frac{10\times 10^{-6}[\text{m}]\times 4.3\times {\left(86400\right)}^{-1}\left[\frac{\text{m}}{\text{s}}\right]}{10^{-9}\left[\frac{{\text{m}}^{2}}{\text{s}}\right]}≅0.5$. This gives the Sherwood number as $\text{Sh}=\frac{1}{2}(1+{\left(1+2\times 0.5\right)}^{1/3})≅1.13$ indicating a 13% increase in the nutrient uptake rate.

As a rough estimate, it can be demonstrated that the ratio of the Sherwood number to the dimensionless relative flagellar cost $C_{\text{rel}}$_._ (which includes construction and operating costs) yields the relative growth rate, $\mu_{\text{rel}}$_._, under nutrient-limited conditions [21]:

(9) $$\mu_{\text{rel}.}=\frac{\text{Sh}}{1+C_{\text{rel.}}}.$$

According to the estimate outlined in Equation (9), eukaryotic flagellated microswimmers with a volume exceeding 1000 μm^3^ encounter an increase in their growth rate, while those with a volume less than 100 μm^3^ find nutrient gathering by flagella to be uneconomical, since its cost outweighs its benefits [21]. This suggests that there might be other benefits to employing flagella in relatively small species, besides enhancing nutrient uptake rate.

Research indicates that collaboration between a large host cell, such as the *Coscinodiscus wailesii* diatom, and numerous smaller epibionts, such as the *Pseudovorticella coscinodisci* ciliates attached to and residing on the host cell’s surface, significantly boosts nutrient absorption by sinking diatoms [111]. This synergy can enhance nutrient uptake by four to tenfold. Moreover, as the quantity of epibionts rises, the fluid flows generated by these auxiliary cells elevate the Sherwood number of the host diatom [111]. The associated costs of this collaborative effort for both diatoms and ciliates remain unknown.

Research has demonstrated that cell-cell hydrodynamic interactions in a large suspension of swimmers enhance the nutrient uptake rate of individual species [112]. The following equation links the Sherwood number before (Sh_0_) and after (Sh) hydrodynamic interactions. “Before” refers to swimming in a dilute limit where hydrodynamic interactions are negligible, while “after” pertains to conditions where the volume fraction of swimmers (the volume occupied by swimmers relative to the total volume of the confined environment) is increased [112]:

(10) $$\tau_{e}^{\prime }=\frac{\text{Pe}}{3\phi Sh}\approx \frac{\text{Pe}}{3\phi Sh_{0}\left(1+0.61\phi +4.7\phi^{2}\right)}$$

where $\tau_{e}^{\prime }$ and ϕ represent the dimensionless relaxation time (defined as the time for the relative nutrient concentration —current concentration divided by initial concentration— to decrease to 1/e) and the volume fraction of swimmers, respectively. When Equation (10) is applied to the model coccolithophore *Calcidiscus leptoporus*, with the previously estimated Peclet (~0.5) and Sherwood (~1.13) numbers, and considering a volume fraction ranging from 0.1 to 1, the revised Sherwood Number resulting from hydrodynamic interactions will vary between 1.25 and 7.13, while the relaxation time will range from 1.33 to 0.02.

### 5.2. Cost Analysis

Motility costs consist of any costs associated with motility from the time the organism starts moving due to a motive until it stops. These include: 1) the power required for swimming and sinking/rising, determined by the drag force exerted on the organism due to viscosity and pressure; 2) the cost of constructing and operating motility-related machinery, such as visible structures like flagella or non-visible components found in cyanobacteria, diatoms, etc.; 3) the cost related to the efficiency of motility-related internal machinery and external organism shape; 4) the expenses related to morphological adjustments; and 5) costs related to migration mechanisms, including strategies for navigating turbulence and predator-prey interactions. Some costs are one-time expenses measured in joules or ATP per cell, while others are continuous costs measured in joules or ATP per second per cell. The Gibbs free energy generated by ATP hydrolysis varies significantly, from 28 kJ/mol under standard conditions to up to 55 kJ/mol, as observed in *E. coli* during exponential growth phases [113].

It is important to note that all these processes are time-dependent. While the cost of swimming at a relatively slow speed or for a short duration might be relatively economical compared to the microorganism’s metabolic rate, it can become significantly expensive for faster speeds and longer durations of swimming under turbulence. This is because, firstly, the actual metabolic rate fluctuates during the organism’s migration, influenced by factors such as activity level, nutrient availability, temperature, and light conditions. Secondly, the motility of organisms, as observed in diatoms [34], may not be steady, leading to time-dependent energy dissipation—an inherent property of non-equilibrium systems.

#### 5.2.1. Construction Cost of Flagellum

One notable expense associated with the phytoplankton swimming process is the construction cost of the flagellum. While it might be assumed that this is a one-time occurrence and therefore not a substantial energy fraction over the lifespan, research indicates that microorganisms can lose their flagella either accidentally or through programmed mechanisms. For instance, bacteria have the ability to remove or deactivate their flagellum in conditions of nutrient starvation [114,115]. Consequently, it is important to account for a flagellum loss rate when studying phytoplankton swimmers.

The construction cost refers to the expenses associated with building the proteins and lipids necessary for the formation of a flagellum. This is calculated by considering the number of proteins, the length of the proteins in terms of amino acids, and the average energy cost per amino acid as proposed by Schavemaker and Lynch [21]. Additionally, since eukaryotic flagella are enclosed by a membrane, the construction cost of the membrane should also be included. According to the estimation provided by these authors, without accounting for flagella loss rate, the costs of constructing flagella relative to the cost of constructing the whole cell in eukaryotes, assuming a spheroidal cell shape, are suggested to range between 0.037% and 44%, with a median value of 3% [21]. The absolute cost of constructing flagella rises with cell volume because the total flagella length (product of the number and length of flagella) increases with cell volume, albeit with varying exponents among different groups. However, relative to the total construction cost of the cell, the cost of flagella construction tends to decrease as cell volume increases [21].

The cost associated with any modification to the flagellum, such as adding a single amino acid to the protein’s structure, varies depending on the type of protein involved. This range extends from the relatively low cost of inner arm dyneins to the higher cost of tubulins, in relation to the overall cell construction cost [21]. The cost assessment of modifications in the flagella is important because the length of flagella varies within-cell due to biological fluctuations connected with LF genes (specifically LF1 [116,117]), which regulate the flagellum length-control machinery and lead to the addition or removal of a large number of tubulins [116]. The system regulating flagellar length in *Chlamydomonas reinhardtii* displays fluctuations significantly greater than measurement error, with a variability coefficient ranging from 10% to 20% [116]. Therefore, the cost associated with these within-cell flagella length fluctuations [116], which involve the removal and addition of amino acids from/to proteins constructing the flagella, is important, as estimated in [21]. However, there should always be a break-even point where further increases or decreases in flagella length become ineffective for the cell. The length should be adjusted to maintain a balance between costs and benefits, ultimately leading the cell to adopt an optimal intermediate length.

A microswimmer with a higher construction cost of flagella (due to having more and/or larger flagella) possesses the capability to achieve greater swimming speeds, as the log-log experimental data shows (albeit with a low R^2^ ~ 0.35) [21], leading to increased drag force and power consumption in a viscous environment.

#### 5.2.2. Construction cost of Non-visible Swimming/Sinking Machineries

Cyanobacteria such as *Synechococcus* and *Synechocystis sp.* swim using non-visible machinery including protein motors that generate traveling waves on the cell membrane, but little is known about the construction cost of the components involved in the propulsion of these organisms. Also, the sinking of phytoplankton such as diatoms is often considered a passive process, yet these microorganisms actively employ strategies to regulate their sinking/rising behavior and speed and little is known about the construction expenses for the machinery involved in regulating buoyancy. Here, we provide a biological overview of the construction cost of these non-visible motility apparatus. However, precise estimation of the associated costs remains a subject for future studies.

The essential components for the swimming of *Synechococcus* include the crystalline S-layer (surface layer) [118] composed of elongated subunits tilted approximately 60° with respect to the cell wall, SwmA glycoproteins [119], SwmB polypeptides [120], and protein motors associated with the peptidoglycan layer [53]. To estimate the construction cost of these motility-related components, one needs to determine the number and length of the subunits of the S-layer, SwmA, SwmB, and motor proteins. To the best of our knowledge, the copy number of all these components is not available in the literature.

The construction cost of motility-coupled components in *Synechocystis sp.* is linked to the copy number of type IV pilus (T4P) machines. These machines are composed of major and minor pilins, secretin, alignment proteins, platform proteins, assembly ATPase, and retraction ATPase [57]. To estimate the construction cost of the T4P in *Synechocystis sp.*, we refer to the PDB ID 3JC8, which represents the “Architectural model of the type IVa pilus machine in a piliated state” of *Myxococcus xanthus* [121]. This structure, determined using electron microscopy, consists of 37,468 amino acid residues [121]. Recent measurements indicate that the average copy number of T4P machines in *Synechocystis sp.* varies between 15 and 20 per cell [122]. Constructing a single amino acid requires an average of 29 ATP molecules [21]. Using these figures, we can calculate the average construction cost of the T4P machines as follows: 20 [machines] × 37,468 [residues per machine] × 29 [ATPs per residue] = 21,731,440 ATPs. This is approximately 2.2×10^7^ ATP molecules. Given the potential differences in the number of residues between T4P of *Myxococcus xanthus* and *Synechocystis sp.*, we also propose an alternative approach. We use the reported size of T4P machines in *Synechocystis sp.* [122] to estimate its volume. Using the average volume per amino acid residue (1.33 × 10^−10^ μm^3^ [21]), we can then determine the number of residues per T4P machine. The average length and width of T4P machines in *Synechocystis sp.* are reported to be 2.5 μm and 0.007 μm, respectively [122]. Assuming the T4P structure is cylindrical, this gives an approximate volume of 9.6 × 10^−5^ μm^3^. Dividing this volume by the average volume per amino acid residue results in approximately 7.2 × 10^5^ residues per T4P machine in *Synechocystis sp.*, which is about 20 times more than the residue count derived from the PDB ID of *M. xanthus* T4P. Therefore, the average construction cost of the T4P machines in *Synechocystis sp.* can be calculated as: 20 [machines] × 7.2×10^5^ [residues per machine] × 29 [ATPs per residue] = 4.2×10^8^ ATPs. Assuming that T4P machines are the sole contributors to motility in *Synechocystis sp.*, our estimated construction cost aligns with the median construction cost of all flagella of bacteria as reported in [21].

The construction cost associated with buoyancy regulation in phytoplankton varies with the number and size of proteins associated with the synthesis of gas vesicles in cyanobacteria [123], the need to syntheses organic osmolytes [80], the construction cost of pores and channels that mediate the active influx of water from the cytosol into the vacuole [80], and the replacement of heavy ions with light ions in vacuole [69,80]. Also, in diatoms, the energy investment in frustule formation, which is strongly related to sinking, entails silica precipitation, cytoskeletal organization of silica-rich vesicles, silica metabolism regulated by genes, and assembly costs [67,69]. The calcification and formation of the coccolith layer in coccolithophores play a similar role in contributing to the sinking patterns of these microorganisms [124]. One challenge in determining the construction cost of the components regulating phytoplankton buoyancy is that these components serve multiple purposes, making it difficult to estimate the fraction of energy expenditure specifically related to buoyancy regulation. For gas vesicles to provide buoyancy, 3 to 10 percent of the cell volume should be occupied by gas vesicles, depending on their mass density [123]. Approximately 10% of this volume is composed of the protein wall, which has a mass density of around 130 kg/m^3^, while the remaining volume is gas space, with a mass density of about 1 kg/m^3^ [123]. According to estimates by Walsby [123], the construction cost of gas vesicles in *Escherichia coli* to achieve neutral buoyancy is approximately 2.84×10⁻¹⁰ J, which is roughly equivalent to the energy content of 3.11×10⁹ ATP molecules.

### 5.2.3. Swimming External and Internal Efficiency

Swimming efficiency is described as the ratio of the power used for propulsion over a specific distance to the power required to pull the organism through the viscous fluid. The swimming efficiency of flagellated unicellular organisms, whether eukaryotes or bacteria, is generally below 3% [109,125–131]. Swimming efficiency can be divided into external and internal efficiency. External efficiency encompasses energy dissipation related to the swimmer’s external characteristics, such as body geometric shape [18], flagellum-to-body length ratio [109,131], and flagellum slenderness [109,131], all of which are affected by the hydrodynamic viscosity of the environment. In contrast, internal efficiency pertains to energy dissipation within the organism, such as the elastic bending of the axoneme [22,132]. The external efficiency associated with the physical properties of flagella has been reported to be 0.8% for the green alga *Chlamydomonas* [109] and 1.4% for *Tetraflagellochloris mauritanica* [131].

As described above, the external efficiency depends, in part, on the shape of the microswimmer. Ignoring the trade-off between microswimmer shape, navigation cost, and optimal navigation time [133], higher external efficiency values are observed for prolate-shaped microswimmers. These values decrease as the shape transitions towards more compact forms (such as spherical or cubic), with the lowest values found in oblate shapes [18,134,135]. The maximum external efficiency or minimum dissipation for various geometric shapes is determined by the following formula. In Equation (11), 𝑅_PS_ represents the drag coefficient of a perfect-slip rigid body, while 𝑅_NS_ denotes the drag coefficient of a no-slip rigid body [18]:

(11) $$\xi_{m}\leq 1-\frac{R_{PS}}{R_{NS}}$$

The theoretical model formulated by Giri and Shukla [136] demonstrates that the external power consumption of swimming, with respect to the shape aspect ratio, for self-propelled free microswimmers utilizing tangential surface actuation, exhibits a variation spanning more than six orders of magnitude. We integrate the theory developed by these authors [136] with empirical data on the elongation and aspect ratio of phytoplankton species [137]. For example, we find that the external energy dissipation during swimming in certain Ceratium species, a genus of dinoflagellates characterized by an aspect ratio of 0.025 and oblate elongation, is exceptionally higher compared to certain Closterium species, a genus of Charophyte green algae characterized by an aspect ratio of 112.83 and prolate elongation, differing roughly by six orders of magnitude.

The examination of over 5700 phytoplankton species’ shapes spanning 402 genera reveals they can be categorized into 38 distinct geometric forms [137]. This investigation indicates that intermediate-sized species exhibit the most significant shape variation, while small and large cell sizes tend to be predominantly compact (spherical or cubic). Among the various shapes investigated, the authors reported that 46% are considered prolate, 38% compact, and 16% oblate [137]. This classification is determined by the aspect ratio, which compares the largest and smallest orthogonal dimensions of cells. Prolates have a ratio greater than 1, compact cells have a ratio close to 1, and oblates have a ratio less than 1. A complicated yet valuable endeavor involves applying the theoretical framework pioneered by Guo *et al.* [134] to calculate the external power dissipation during swimming for these 38 shapes [137].

On the other hand, swimming internal efficiency, which is equally significant, refers to the effectiveness of the internal propulsive layer, axoneme, of the phytoplankton swimmer. The primary sources of energy dissipation in the internal propulsive layer are flagellum bending, sliding of microtubules, and internal viscosity [22,132]. Experiments on the ATP consumption rate per beat cycle of the flagellum in *Chlamydomonas reinhardtii* reveal that the energy dissipation due to the elastic bending of the axoneme is an order of magnitude greater than the internal hydrodynamic energy dissipation [132].

The detailed swimming internal efficiency of *Synechococcus* and *Synechocystis* cyanobacteria remains less explored. However, it is evident that any energy dissipation linked with motility mechanisms should be taken into account. For instance, in *Synechococcus*, components such as the S-layer and protein motors moving along helical paths contribute to energy dissipation, while in *Synechocystis*, the type IV pilus could play a similar role. In *Synechococcus* cyanobacteria, the subunits of the S-layer form a two-dimensional lattice with varying spacing, typically ranging from 5 to 22 nm, resulting in oblique, triangular, square, or hexagonal shapes [138]. These structural variations, unique to each species, may lead to different ranges of energy dissipation. This dissipation likely occurs as motor proteins make frictional contact with the S-layer and move beneath it along a helical path, generating traveling waves on the organism’s surface [53]. Since these motor proteins are driven by proton motive force [53], another source of dissipation likely occurs in this process, presumably in the form of proton leakage, as extensively reported in the membrane of other organelles like mitochondria [139–141]. In type IV pilus machines in *Synechocystis*, one reported source of inefficiency is related to the retraction process, mostly in the absence of ATPase [57,142].

It’s worth noting that a low efficiency in swimming doesn’t necessarily equate to a high swimming cost when compared to the total metabolic rate of the cell [125,143]. This needs to be investigated case by case as many parameters are influential as studied in the present article. We will provide an example and discuss this in more detail in the section “Swimming/Sinking Cost to Total Metabolic Rate Ratio.”

#### 5.2.4. Sinking/Rising External and Internal Efficiency

The shape-dependent external efficiency of sinking species appears to be more complex than that of swimmers [77]. Non-spherical sinkers (both single cells and colonies) generally exhibit a slower sinking rate compared to their equivalent spherical counterparts, except for tear-drop-shaped sinkers [19,144]. To the best of our knowledge, there is no solid theory in the literature explaining why the shape-related external efficiency differs between swimmers and sinkers.

The correlation among shape, speed, ocean depth, nutrient concentration, and light availability is significant. For instance, deep waters often exhibit low-light and high-nutrient conditions concurrently. Therefore, it is logical to expect an augmentation in cell elongation with increasing depth, regardless of whether the waters are poorly mixed or well-mixed [137]. This is presumably because phytoplankton with elongated shapes are adept at arranging chloroplasts along their cell surfaces, thereby enhancing light absorption, particularly at greater depths [145]. The geometric shape is also influenced by seasonal variations, with different species of diatoms exhibiting a predominantly oblate shape in spring and autumn and a predominantly prolate shape in summer [146].

The internal efficiency of sinking and rising in phytoplankton, such as diatoms, remains *largely unknown*. This is likely because these species have traditionally been considered passive motile microorganisms in physical models. We propose classifying their motility as active, albeit non-visible. Using this new classification, it is essential to define, calculate, and formulate the internal efficiency of phytoplankton’s sinking and rising movements. This internal efficiency can be defined as any dissipation occurring in contact with the components that facilitate sinking/rising. The buoyancy regulation strategy can be more energy-intensive than swimming, owing to the diverse modulatory processes a cell must undertake to move both downward and upward, involving the construction of heavier or lighter macromolecules depending on the condition [23,147]. According to Raven and Lavoie [23], the energy cost of synthesizing gas vesicles in cyanobacteria is 1.3 × 10⁻³ relative to the total synthesis cost of the cell, whereas the active water influx in the diatom *Ethmodiscus rex* is 0.016 relative to the same.

The major mechanisms facilitating buoyancy regulation in phytoplankton and the possible associated internal energy dissipation are proposed in the following.

1. Gas vesicles, which mediate buoyancy regulation in prokaryotes such as cyanobacteria and haloarchaea, consist of a gas-filled space enclosed by a protein wall primarily composed of proteins like GvpA and GvpC [123,148,149]. The efficiency of gas vesicles in providing buoyancy is proposed to be influenced by the interaction of their geometrical properties, elastic modulus, and turgor or hydrostatic pressure [150]. This efficiency is qualitatively defined by the volumetric ratio of the gas space, $V_{i}$, to the surrounding proteins, $V_{w}$, which indicates how much buoyant force is generated relative to the structural material [150]. As the radius and length of the cylindrical-shaped vesicle increase, the efficiency improves because a larger gas volume provides more buoyancy with relatively less protein weight. However, this improvement in efficiency eventually saturates with increasing length [123,150]. The shape of gas vesicles changes over time, starting as small biconical structures at formation and developing into cylindrical and spindle shapes at maturity [149]. Under normal conditions, gas vesicles can achieve efficiencies as high as 90% of the theoretical maximum, defined as $\frac{{\left(\frac{V_{i}}{V_{w}}\right)}_{\text{measured}}}{{\left(\frac{V_{i}}{V_{w}}\right)}_{\text{theor.max}.}}\times 100$, formulated for infinitely long gas vesicles [150]. However, changes in hydrostatic pressure, such as those experienced in deep ocean environments or under high light intensity, can reduce this efficiency and may cause gas vesicles to collapse, disrupting buoyancy regulation in cyanobacteria [151]. If the cell survives, reforming gas vesicles is a resource-intensive process [151]. The ability of gas vesicles to withstand pressure depends on the elastic modulus of their structure, necessitating a mechanical analysis of stress and strain [152].
2. Buoyancy regulation in diatoms is managed by balancing protoplasm density adjustments and the silicification process in the frustule. The mass density in both the protoplast and frustule can be either increased or decreased relative to water. Optimal buoyancy regulation occurs when these two density adjustments align positively, meaning both compartments either simultaneously increase or simultaneously decrease their density relative to water. Conversely, if one compartment increases density while the other decreases it, this misalignment reduces efficiency and increases energy dissipation. The density of protoplasm is changed through the processes like the synthesis of organic osmolytes and trimethyl ammonium [23,62,147], and the active influx of water from the cytosol into the contractile vacuole [80,82] while the density of frustule is changed through silicification [67,153]. The silicification process generally leads to an increase in frustule density [69]. However, it has been reported that ocean acidification can significantly reduce silica production [154]. Additionally, bacterial attack on diatoms using their hydrolytic enzymes increases the rate of silica dissolution, resulting in a lower mass density of diatoms [155,156]. Other possible sources of dissipation might involve cell expansion, which leads to mechanical deformation and the release of elastic energy during density changes. This process is also associated with the relative movement of the valves of the frustule, potentially contributing to hydrodynamic dissipation. Given the rigidity of the frustule and the average force required to break it (0.2 – 0.8 mN [157]), any deformation can be energetically costly. The leakage of solutes through the membranes (tonoplast and plasmalemma), associated with changes in the density of the vacuole and the cell wall, is another source of energy dissipation [81].
3. Calcification associated with the calcified shells (coccospheres) of coccolithophores significantly alters their sinking rates. For instance, in small *E. huxleyi*, which ranges from 4 to 9 μm in size, calcification changes the sinking speed from 3 cm/d to 30 cm/d—an order of magnitude difference between naked and calcifying organisms [124]. While the literature primarily examines the role of calcification in increasing density and sinking rates in coccolithophores [124,158], these organisms can also regulate buoyancy by stopping calcification under extreme light-limited conditions, thereby lowering cell density [159,160]. However, under nutrient-limited conditions, calcification continues, allowing the dissipation of absorbed light energy since cell growth is restricted by the lack of nutrients [159,161]. Possible sources of hydrodynamic dissipation may also exist within the cell-wall layer, involving the interplay of coccoliths, body scales, and baseplate scales during the calcification process and density adjustment. The coccosphere has multiple roles beyond buoyancy regulation, including mechanical protection, as evidenced by its strong inelastic tolerance to forces [162], protection against photodamage [163], and acceleration of photosynthesis [124]. These diverse roles complicate the assessment of the cost of calcification associated with internal buoyancy-related mechanisms. One approach to assess the role of calcification in buoyancy regulation is to measure the oxygen consumption rate under dark conditions when the phytoplankton is both suspended and sinking. This can be achieved using optical fluorescence oxygen sensors [42] or modern methods that measure extracellular acidification and oxygen consumption rate, providing ATP turnover within the cell [164].

The fluctuating sinking and rising behavior, characterized by periodic (~ every 10 seconds) rapid bursts of speed followed by slow, near-zero movement observed in diatoms [34,78], may significantly contribute to elevated energy expenditures [82]. Lavoie and Raven have estimated that the minimum energy expenditure for this behavior accounts for 16% of the total energy cost of growth. This is achieved through the modulation of sodium and potassium permeability, interconversion of low-density and high-density organic cations, and alterations in cell expansion rate [82]. The rapidly fluctuating processes are recognized for their high energy dissipation rates in nonequilibrium thermodynamics [165], a factor that should be considered in future analyses. We suggest that this unsteady sinking behavior can be mathematically simulated by incorporating a stochastic term into the buoyancy equation (Equations (2) or (3)). This stochastic term, representing a time-dependent ‘burst noise,’ should be defined by a base noise level, a burst amplitude, and a random component, and should be multiplied by the organism’s mass density.

If the fluctuating sinking and rising behavior results from cell expansion, it could be due to fluctuations in organelle size, such as those in vacuoles. It has been reported that cells have robust mechanisms to control these fluctuations in organelle size [166]. Do diatoms have any control protocols to minimize energy dissipation during rapid changes in their sinking or rising speeds, even though the control process itself expends energy [167]? Here, we propose two basic ideas regarding the physics of unsteady buoyancy regulation in terms of cell expansion and jumping process.

To adjust its internal density, an organism needs to change its mass and volume so that their ratio increases (to move downward) or decreases (to move upward) relative to the density of water. This rapid and sudden change in mass and volume is analogous to the inflation and deflation of a balloon. When inflating a balloon by mouth, the initial burst of air is typically followed by a pause before the next round of blowing. Classical physics shows that the first attempt to increase the balloon’s radius requires much more pressure than subsequent inflations [168,169]. This is due to the resistance of the polymer chains’ chemical bonds in the balloon until a threshold stretch is reached [170]. Similarly, in species that exhibit rapid bursts of speed when sinking or rising, the initial sudden effort to increase/decrease the mass density and consequently the speed may dissipate much more energy compared to subsequent efforts. This hypothesis could be tested in future experimental studies. Fundamental physics also tells us that the time it takes for a balloon to deflate is related to the radius of the balloon by the equation $\tau ∼R_{B}^{7/2}$ [171]. Can this method also be applied to phytoplankton that regulate buoyancy?

The rapid alterations in diatoms’ motility speed, prompted by sudden shifts in mass density, might be analogous to the leaping or jumping motion observed in various creatures such as frogs or insects the fundamental physics of which has been investigated extensively [172–175]. As these organisms jump, the elastic energy from morphological changes and shifts in mass density dissipates through interaction with the viscous water surrounding them. Consequently, by considering the forces at play, one can estimate both the distance covered during each jumping-like speed change and the hydrodynamic dissipation involved.

#### 5.2.5. Predator and Virus Risk

Swimming and sinking expose phytoplankton to potential predation risk due to the flow fields they generate and the deformations they induce in the fluid, which can serve as signals for surrounding predators [176]. In nature, these signals are physical quantities of information. Motile organisms capture and assess this information content, expending energy measured in $k_{B}T$ per bit [177], where $k_{B}≅1.38\times 10^{-23}$ [J.K^−1^] represents the Boltzmann constant, and T denotes absolute temperature [K]. The information-processing cost depends on the rate at which the organism receives and processes information, which varies according to the condition. While the information processing cost by the motility machinery might be negligible compared to other motility costs, it is important to understand how organisms recognize and distinguish between signals (information) received from prey or predators and those generated by fluid disturbances caused by turbulence. There might be a way for organisms to identify information from different sources, which is essential for determining their strategy before it’s too late. This is where the concept of the “value of information” becomes relevant, a largely forgotten concept developed by Volkenstein [178] and recently revisited (section 14 of [179]). The value of information is defined by its irreplaceability or non-redundancy as the logarithm of the ratio between the probability of an outcome after receiving information and the probability of that outcome before receiving information. Recognizing the value of information may provide organisms with a competitive advantage in the course of evolution [178].

It has been proposed that the risk of mortality for microorganisms is a function of the product of their size and swimming or sinking speed, particularly when speeds exceed 50 μm/s [20]. This product reflects a balance between two factors: the benefit of increased clearance rate, which improves resource acquisition, and the increased vulnerability to predation due to a larger cross-sectional area. At these higher speeds, the trade-off between these opposing factors becomes a useful measure for assessing the likelihood of mortality [20]. Increasing the clearance rate correlates with elevated mortality risk, particularly pronounced among quadrupole swimmers (defined as swimmers with two opposing equal time-averaged point forces, lateral flagella, and an opposing point force with double the magnitude applied at the origin to represent the cell body – Table 1), who face the highest predation risk. In contrast, stresslet swimmers (defined as swimmers with one flagellum represented as a point force, with an opposing equal point force representing the cell body) experience the lowest predation risk. Although dinoflagellates are classified as quadrupole swimmers based on force arrangement (as indicated in Table 1), their categorization shifts to stresslet swimmers concerning noise levels. The presence of both a transversal and trailing flagellum enables them to exhibit stealth behavior, even at high swimming velocities [20].

How does prey-predator interaction influence the motility costs of both organisms? The direct cost arises from the increased speed required for evasion, pursuit, or escape, leading to higher swimming or sinking power consumption as described by Stokes’ law [41]. Indirect costs stem from defense or attack mechanisms, such as the production of toxic substances, which can impair motility machinery or reduce growth rates. The concept of indirect cost arises from the fact that defense mechanisms force the organism to allocate energy toward producing toxic substances, reducing the energy available for other cellular processes and increasing the ratio of motility cost to total metabolic rate. For example, theoretical models for dinoflagellates show that producing toxins can significantly reduce the rate of cell division—by over 20%—especially under nitrogen-starved conditions [12,31]. Defense costs may be lower owing to the inherent cell-wall structure of the prey. For instance, copepods efficiently hunt thin-shelled diatoms, yet they frequently reject thick-shelled ones [180]. These instances contribute to the mortality rate due to the inherent predation risk.

Prey can utilize another defense mechanism through the production of blue bioluminescence [181]. This process incurs a high energetic cost due to the chemical reaction between luciferin substrate and luciferase enzyme in the presence of oxygen [9,182]. Bioluminescent dinoflagellates, which produce 10^7^ to 10^11^ photons per flash, possess an extraordinary capability to communicate with other organisms up to 5 meters away [11,181,183]. To put this in perspective, it’s like a person standing at a height of 2 meters effectively conveying a message across a distance of 20 kilometers [181]. Research indicates that copepods, which prey on dinoflagellates, utilize high-speed swimming bursts to avoid dinoflagellates when exposed to bioluminescent light, resulting in a reduction in dinoflagellate mortality rates [184].

The formation of chains and colonies can either heighten or diminish predation risks depending on conditions. For instance, studies indicate that diatoms often aggregate into chains during blooming periods and in cooler climates, yet they typically remain solitary in warmer environments with lower population densities [185]. Conversely, when encountering copepod grazers, diatoms have been observed to reduce their chain length [186].

The size and speed of microswimmers/sinkers can significantly influence the likelihood of viral infection [187]. Studies on flagellated bacteria have shown that while swimming may increase the susceptibility to viral infection, hydrodynamic interactions mitigate this risk around the cell body while exacerbating it near the flagellar bundle [188]. This variation stems from differences in fluid flow dynamics around the flagella compared to those around the cell body [188]. Furthermore, experiments involving ciliates fed with various viruses, including mammalian viruses and bacteriophages, indicate that the presence of phages progressively enhances the swimming speed of ciliates [32]. However, the costs and benefits associated with viral infection on the swimming or sinking behavior of phytoplankton remain unknown.

#### 5.2.6. Migration cost

If one possesses knowledge of the swimming speed and the characteristic size (which considers factors such as shape, and flagella properties, etc.) along with the viscosity, they can be used to compute the power consumption over extended distances or periods, assuming the swimming cost relative to the total metabolic rate remains constant. Another significant parameter proposed is the chaoticity of the migration path, which appears to play a considerable role in energy expenditure [29]. It is estimated that the cost of the vertical diel migration of diatoms is negligible compared to their daily net photosynthetic production [29]. However, it’s crucial to note that the total metabolic rate is not constant during migration due to varying factors such as nutrient availability, light intensity, and temperature, all of which are depth-dependent. Consequently, the swimming/sinking speed fluctuates significantly during migration, potentially resulting in higher relative costs under nutrient and/or light limited and/or turbulent conditions. The other parameters involved are both internal and external efficiency of motility as discussed in previous sections.

The time-dependent costs incurred during migration, whether depth-dependent or strategy-dependent, are typically overlooked in cost estimations. It’s evident that various parameters vary according to depth [25]. Additionally, under certain conditions, phytoplankton exhibit different strategies. For example, the associated costs of chain formation can include resource sharing between neighboring cells and the cost associated with chain rigidity and breakage [185]. Furthermore, the cost linked to dividing migrators into two subpopulations to evade turbulence, as discussed in previous sections, includes the production of NO as a cellular stress and the obligatory downward movement, which alters the rate of photosynthesis under regions with lower light intensity [30]. These are just a few examples of the inherent costs associated with migration. In the next section, we will discuss and estimate an example of the comprehensive costs associated with motility. Table 2 provides a summary of all the motility costs discussed in this paper.

### 5.3. Swimming/Sinking Cost to Total Metabolic Rate Ratio

To determine what percentage of the total metabolic rate of the cell is accounted for by the swimming/sinking power consumption, one requires data on the total metabolic rate of the studied species. This task is more straightforward for heterotrophic protists under growing or starved conditions, as it can be obtained through the rate of oxygen consumption [41,189,190]. However, this poses a greater challenge for phytoplankton cells, whether they are photoautotrophs or mixotrophs, because these microorganisms are both producers of oxygen in their chloroplasts and consumers of oxygen in their mitochondria and cytoplasm [191]. Recent findings highlight the contrasting metabolic patterns per mitochondrion relative to cell volume between microalgae and heterotrophic protists under dark conditions, underscoring the difference between phototrophic and heterotrophic cells [192]. This challenge highlights the importance of comparing motility power consumption with respiration and photosynthesis rates under both dark and light conditions [191,193–198]. Additionally, it involves comparing motility-related construction costs with the total chemical energy content, including protein, lipid, and carbohydrate content, under various nutrient and light conditions [191,193–198].

We evaluate the potential costs associated with phytoplankton motility using the dinoflagellate *Prorocentrum mariae-lebouriae* as an example. This species is currently considered a synonym of *Prorocentrum minimum* (Pavillard) J.Schiller [199]. *Prorocentrum minimum* is a geographically widespread, bloom-forming dinoflagellate linked to harmful algal blooms, ecosystem damage, and potential shellfish toxicity, often associated with coastal eutrophication [200]. This dinoflagellate measures 14.8 μm in length, 14.8 μm in width, 7.3 μm in depth, and 5.85 μm in equivalent spherical radius, with an oblate shape and an aspect ratio of 0.49 [201]. It has a volume of 846 μm^3^ and a mass density of 1064 kg/m^3^, resulting in a total body mass of approximately 0.9 ng and a swimming speed of 170 μm/s at 20°C [201]. A dinoflagellate with a volume of 846 μm^3^ has a total chemical energy content (comprising proteins, carbohydrates, and lipids, which determine the internal nutrient status of the cell) of 5.3 μJ/cell. This value is derived using the equation log_10_ (energy content [nJ/cell]) = 2.88 + 0.91 log_10_ (volume [μm^3^]/100) from reference [194] developed for dinoflagellates.

We recently formulated an equation to estimate the total ATP production rate in actively growing phytoplankton [14]. Our model incorporates critical parameters, including the dark respiration rate of actively growing cells derived from body volume or dry mass, mitochondrial proton leak (generally ranging from 20% to 50% of the standard metabolic rate [139,202]), the respiration-to-photosynthetic rate ratio (ranging from 0.08 to 0.6 in actively growing dinoflagellates [13,203]), ATP yield per oxygen molecule consumed in mitochondria (which is determined by the carbon source, chemical pathways, and the 10 c-subunits in the mitochondrial ATP synthase of unicellular eukaryotes, resulting in a real value ranging from 2.77 to 4.98 [204]), ATP yield per oxygen molecule produced in chloroplasts (also influenced by the electron transport chain pathways and the number of ATP synthase c-subunits) [205], and ATP generated through cyclic and pseudocyclic photophosphorylation [206]. Additionally, the energy content of hydrolyzing one mole of ATP varies from 28 kJ/mol under standard conditions to 55 kJ/mol as observed in *E. coli*, depending on the pH and the concentration of magnesium ions, as Mg^2+^ increases the stability of ATP molecules by binding to phosphate [113]. Applying this approach to *P. minimum*, with a cell volume of 846 μm^3^, yields total ATP production rates ranging from approximately 15 pW/cell to 210 pW/cell or 3.2×10^8^ ATPs/cell/s to 2.3×10^9^ ATPs/cell/s at 20°C. The total metabolic rate estimated here varies depending on the range of values assumed by the involved parameters, meaning the result can lie anywhere within these extremes based on physiological conditions. Given the global ocean temperature range of −1.8°C to 30°C, these values are expected to vary between approximately 3.3 pW/cell or 7.1×10^7^ ATPs/cell/s at −1.8°C as the lower bound and 420 pW/cell or 4.6×10^9^ ATPs/cell/s at 30°C as the upper bound, assuming a Q_10_ value of 2.

As described by Equations (4) and (5), light intensity decreases exponentially with depth from the ocean surface, while nutrient concentration increases exponentially from ocean surface to the chemocline layers [33,63]. More specifically, the concentration profiles of chlorophyll, nitrogen, phosphorus, and carbon vary with depth [25]. Because migration takes time, time-dependent models can offer insights into the chemical energy content of phytoplankton, from protein, RNA, and chlorophyll concentrations to nitrogen storage, during periods of starvation [207]. Under nutrient limited conditions, the respiration rate also decreases compared to the active physiological state of the cell [189,190,192]. Thus, phytoplankton may partially or fully compensate for nutrient and light limitations through swimming or buoyancy regulation, as estimated by the Sherwood number.

Using the relationship between swimming external efficiency and the shape aspect ratio developed in [18], the aspect ratio of 0.49 for *P. minimum* results in an external efficiency of 0.16. This means the Stokes drag force acting on this organism is 2.08 times greater than that of an equivalent spherical body. This factor is calculated using the ratio $\frac{1}{3}÷\frac{16}{100}$ , as the external efficiency of a spherical body is $\frac{1}{3}$, as obtained in [18]. For external efficiency related to the physical properties of flagella, such as their length, slenderness, and relative angle with the body, we consider the average efficiency reported for green algae [109,131] to be 1%. This indicates that for every 100 units of energy, 99 units are dissipated, meaning the organism must expend 99 times more energy to compensate for the energy required for swimming. Consequently, the swimming power consumption must be multiplied by 100. Additionally, the average microscale viscosity at the cell surface of plankton is approximately 2.6 times the viscosity of seawater, as reported in [28]. Therefore, the power consumed during swimming can be calculated as follows: $P=\text{external dissipation}\times 6\pi \eta rv^{2}=2.08\times 100\times 6\pi \times 2.6\times 0.00109$ [kg.m^−1^s^−1^] × 5.87 × 10^−6^ [m] × (170 × 10^6^)^2^ [m^2^s^−2^] ≈ 1.9 pW, where 0.00109 [kg.m^−1^s^−1^] is the bulk viscosity of seawater at 20°C.

Next, we consider the elastic cost of bending the axoneme which is associated with the swimming internal efficiency [22,132]. As reported in [132], the energy expenditure of the axoneme per beat of a demembranated eukaryotic flagellum is approximately 2.3×10^5^ ATP molecules, which translates to 1.41×10^−2^ pJ energy content on average. Assuming that dinoflagellates have two flagella with a beat frequency of 64.8 s^−1^ [20], the bending cost of the flagella is: 1.41×10^−2^×2×64.8 = 1.83 pW. Adding this to the external power consumption due to drag force gives: 1.9 + 1.8 = 3.7 pW. Relative to the total metabolic rate of the species at 20°C (15 pW to 210 pW as estimated above), this ranges between 1.7% and 25%, which is quite considerable. This relative cost is an inherent expense for a species when it decides to move to a new location. In this estimation, we assumed the dinoflagellate relies solely on swimming without buoyancy regulation. However, since dinoflagellates can perform both, if they do so simultaneously, the internal efficiency of mass density adjustment would factor into the above calculations, potentially increasing the overall motility cost. The construction cost of flagella (cc_f_) can be obtained from a fitting equation provided in [21] as: cc_f_ = 2.6×10^10^volume^0.31^ = 2.1×10^11^ ATP molecules = 10.8 nJ. As described in previous sections, the length of flagella can vary by up to ±20% due to fluctuations in the removal and addition of amino acids and proteins. Consequently, the construction cost ranges between 1.6×10^−3^ and 2.4×10^−3^ times the total chemical energy content of *P. minimum* (5.3 μJ/cell, as estimated above).

Let’s now consider other potential costs associated with motility, particularly those incurred during migration, for the species considered in this example: a dinoflagellate. The migration path is not a straight vertical line but rather chaotic, resulting from the random-walk-type motility of microorganisms [29]. The expected vertical path corresponds to the displacement, while the actual chaotic path corresponds to the distance of migration. For instance, if the chaotic distance is ten times the vertical displacement, the energy expenditure over migration should be multiplied by ten.

Dinoflagellates actively respond to turbulent conditions using various strategies, including density adjustment, shape modification, and changes in orientation [30]. These dynamic changes affect swimming power consumption, as they are directly related to both speed and the swimming internal and external efficiencies. The density changes in dinoflagellates are proposed to be mediated by the cell nucleus, with a mass density of 1300 kg/m^3^, and by 15–20 chloroplasts, each with a mass density of 1150 kg/m^3^ [30]. The actual costs associated with these rapid changes under turbulence remain unknown, representing a gap in our knowledge. However, it has been observed that the rate of stress, measured as the rate of production of the nitric oxide (NO) free radical under turbulent conditions, increases [30]. Estimating the energetic cost of NO production and its accumulation in the cell constitutes the second gap for future studies, even though the mechanisms involved in the production of this radical in living systems are known [208].

For all other occurrences, including but not limited to predation risk, viral infection, chain formation, and colony formation, it is important to consider how these events affect motility speed or the efficiency of both external and internal motility mechanisms.

## 6. The Interdependence Among Viscosity, Size, Speed, and Temperature

The projections regarding the future of climate change and global warming raise several concerns, one of which revolves around the fate of marine phytoplankton and their role in carbon cycling within the ocean [209–216]. Establishing a mechanistic model that links swimming or sinking velocities to temperature variations appears complicated, given the myriad of effective parameters discussed in previous sections. In a recent study [217] on phytoplankton behavior, researchers proposed that movement towards warmer locations, rather than nutrient availability, was the primary driver for the development or maintenance of motility in these organisms. This is because higher temperatures increase metabolic rates, enhancing the energy available for movement, while simultaneously lowering water viscosity, making locomotion energetically cheaper. Additionally, warming intensifies stratification by creating stronger temperature gradients in the water column, which limits vertical mixing and leads to nutrient-depleted surface layers. Under these conditions, motility becomes a crucial adaptive trait, enabling organisms to actively navigate between nutrient-rich deeper layers and light-rich surface waters. Interestingly, the study found that seasonal temperature variations did not significantly influence motility [217], suggesting that the effects of sustained warming differ fundamentally from natural seasonal changes. Additionally, elevated CO_2_ levels have been demonstrated to reduce the motility speed of flagellated microalgae over the long term [218]. On top of these temperature and CO₂ effects, evolutionary and community composition changes can further influence motility patterns over time.

Each enzymatic reaction rate follows an exponential temperature dependence as described by the Arrhenius law. However, because the Arrhenius law does not always fully capture the temperature dependence of a system, other models have been developed. These include quasi-equilibrium transition-state theory (TST) [219], which uses Gibbs free energy of activation instead of activation energy and a theoretically derived pre-exponential factor, and non-equilibrium TST [220], which accounts for the stochastic nature of the system. Additionally, empirically-fitted models introduce new parameters, such as the deformation parameter (which measures deviations from the Arrhenius law) and temperature-dependent activation energy [221].

External, environmental, parameters, such as sea water viscosity and density, along with hydrodynamic interactions [222], are all contingent on temperature, consequently impacting phytoplankton’s swimming or sinking speed. Internally, a cascade of events occurs in response to temperature changes. Initially, a temperature gradient is sensed, likely through ion channels [223], followed by temperature-dependent alterations in the surface tension [224,225], elasticity [225–227], and permeability [228] of the cell membrane, leading to changes in cell shape and size [146], thereby affecting external efficiency in swimming or sinking.

Assembly and function of flagella [229], the ATP-hydrolysis rate by molecular motors involved in motility [230], and the internal viscosity and density, are all temperature-dependent, influencing internal efficiency in swimming or sinking. Researchers studied how temperature affects the flagella of the algal species *Chlamydomonas reinhardtii* [229]. They found that when the temperature shifts from 20°C to 32°C, the flagella respond differently depending on the specific mutant being observed. This range of responses includes flagella becoming broken down, detached, or malfunctioning, all of which incur energetic costs [229]. Additionally, the model proposed in [231] shows that the temperature dependence of beat frequency of eukaryotic cilia and that of the angular velocity of bacterial flagella are governed by the relationships:

(12) $$f=\sqrt{c_{1}Te^{-T\left(x_{1}+x_{2}\right)}+c_{2}}-c_{3}$$

(13) $$\omega =\sqrt{c_{4}T-c_{5}}e^{-\frac{T\left(x_{1}+x_{2}\right)}{2}}+c_{6}$$

where T represents absolute temperature, f denotes the beating frequency, ω is the angular velocity, $x_{1}$ and $x_{2}$ are temperature-viscosity fitting parameters of the fluid and the cell respectively, and $c_{1,\ldots ,6}$ are fitting constants related to parameters including the amplitude, wavelength, and ATP concentration.

Additionally, temperature dependence extends to factors such as maximum nutrient uptake rate, maximum photosynthesis rate, metabolic rate, and maintenance cost, collectively shaping a new population growth rate [232]. It has been observed that increasing the temperature leads to an increased investment in photosynthesis (in *Prochlorococcus* [233] and *P. shikokuense* [234]), nutrient transporters (in *P. shikokuense* [234] and *S. dohrnii* [235]), repair proteins (in *Prochlorococcus* [233], *P. shikokuense* [234], and *C. reinhardtii* [236]), lipid degradation (in *Chaetoceros sp.* [237]), synthesis of saturated lipids (in *Prochlorococcus* [233], *P. shikokuense* [234], and *C. reinhardtii* [236]), stored lipids (in *T. pseudonana* [238], *P. shikokuense* [234], and *C. reinhardtii* [236]), and total protein content (in *T. pseudonana* [239], *Chaetoceros sp.* [237], and *S. dohrnii* [235]) [240]. Conversely, the investment in rubisco (in *C. reinhardtii* [236] and *Prochlorococcus* [233]), ribosomes (in *Prochlorococcus* [233]), and glycolysis (*Chaetoceros sp.* [237] and *S. dohrnii* [235]) decreases [240]. Also, studies suggest that elevated temperatures can boost mitochondrial oxygen consumption, ATP synthesis, and the production of reactive oxygen species in *Drosophila* and *Bufo bufo* [241,242]. However, it is worth noting that mitochondria have been reported to operate at significantly higher temperatures compared to their surrounding cytoplasm [243–247]. If true, this adds a layer of complexity to understanding the impact of external temperature changes on this organelle.

In conclusion, species must acclimate and adapt to these altered growth temperatures [237,248], incurring costs associated with changes in gene expression specifically in pathways linked to acetyl-CoA [237]. The adaptation and tolerance to relatively higher temperatures also depend on the trade-off between respiration and photosynthesis, as these processes have different sensitivities to temperature changes. This phenomenon is observed, for example, in *Chlorella vulgaris* [249]. The difference in temperature responses between photosynthesis and respiration becomes more pronounced under nutrient limitation. Under these conditions, the activation energy of photosynthesis is approximately three times that of respiration, and both are significantly smaller than their activation energies under nutrient-replete conditions, as observed in *Emiliania huxleyi*, *Skeletonema costatum*, and *Synechococcus sp*. [250]. It’s important to note that the temperature response to each factor varies from species to species [251], further complicating the understanding and prediction of phytoplankton motility responses to climate change.

Based on time-independent equilibrium thermodynamics [252], it is suggested that the temperature dependence of any biological attributes, ranging from nano-scale to macro-scale (where larger scale attributes comprise the sum of smaller scale attributes), can be described by the following master equation, under the assumption that heat capacity remains constant regardless of temperature:

(14) $$Y(T)\approx Y_{0}{\left(\frac{1}{T}\right)}^{-\frac{\bar{\text{Δ}C}}{R}-\alpha }{\text{e}}^{-\frac{\bar{\text{Δ}H}}{RT}}$$

In this scenario, Y(T) represents a variable encompassing rates, time, or transient/steady state/equilibrium conditions, α determines the size-scale of the biological attribute, ΔH denotes enthalpy, and ΔC signifies the heat capacity of proteins. However, it is important to note that this model makes several assumptions, including that biological systems can be approximated using equilibrium thermodynamic principles. In reality, biological systems are highly dynamic, operating under non-equilibrium conditions, where external energy inputs and environmental fluctuations can significantly affect their behavior. As such, the application of this equation to the motility of phytoplankton species poses an unresolved challenge, given the complexity and variability of their adaptive responses to changing environmental conditions.

Beveridge *et al.* [26] introduced a model to describe different scenarios involving the temperature dependency of protists’ swimming speed, influenced by the interplay between temperature-dependent viscous drag and metabolic rate. The formula they have derived for swimming speed integrates Stokes’ law, mass-specific metabolic rate, and the temperature dependencies of viscosity and size, expressed as follows:

(15) $$v=\sqrt{\frac{a_{0}i_{0}{\left[M(T)\right]}^{3/4}{\text{e}}^{-E/k_{B}T}}{6\pi \eta (T)r(T)}}$$

where $a_{0}$ represents the proportion of metabolic rate allocated to swimming, $i_{0}$ is a normalization constant with the SI units kg^1/4^.m^2^.s^−3^, M(T) is the temperature dependence of body mass, derived from the assumption of a 2.5% decrease in cell size per 1°C temperature increase in protists [253], $k_{B}$ is the Boltzmann constant, and E is the activation energy. Their findings suggest that swimming speed escalates with rising temperatures [26], aligning with other research exploring temperature-dependent sinking speeds [144,254]. However, Kamykowski and McCollum’s investigation of dinoflagellates’ temperature-acclimatized swimming speed across 5–38°C under stable nutrient and light conditions reveals a trend where speed increases with temperature until reaching a threshold, beyond which it declines [27]. They report that the temperature range supporting swimming ability is broader than the range permitting growth [27]. In their study, the maximum swimming speed is observed in cells approximately 35 μm in length. This decline in speed at higher temperatures and larger sizes can be attributed to physiological limitations and inefficiencies in motility machinery—factors not accounted for by the Arrhenius equation.

Research on the temperature dependence of the size of bacteria, protists, and metazoa indicates a general trend of decreasing species size linearly (at both the single-cell and colony levels) with rising temperatures [253,255]. Temperature is indeed recognized as a major factor influencing size structure, both directly and indirectly [255]. Although no definitive general theory has been established for the temperature-size rule, several hypotheses have been proposed to explain this phenomenon: First, as temperature rises, the availability of essential resources such as carbon dioxide and dissolved oxygen decreases, while metabolic demand increases. Smaller cells can more effectively absorb and utilize these limited resources due to their higher surface area-to-volume ratio, thereby compensating for the reduced resource availability [253]. Contrary to this hypothesis, some bacteria do not depend on gases for survival. Additionally, nutrient availability is not directly temperature-dependent, as it varies by geographical location—some regions become depleted around 20°C, while others do so near 0°C [256]. Second, from an evolutionary standpoint, smaller cell size facilitates faster reproduction, enhancing survival and competitive advantage [253]. However, this raises the question: why don’t organisms remain small in size permanently? Contrary to these propositions, Maranon *et al.* [257] have reported that there is no overarching law elucidating the impact of temperature on phytoplankton size structure. They emphasize that resource availability serves as a major factor influencing size structure. An example that contradicts Atkinson’s size-temperature rule is the finding that *Thalassiosira pseudonana* diatoms increase in size with rising temperatures in evolved lineages after 300 generations of adaptation [239]. This size increase is hypothesized to enhance lipid content, serving as a strategy to mitigate oxidative stress induced by high temperatures [240].

**Figure 1:**
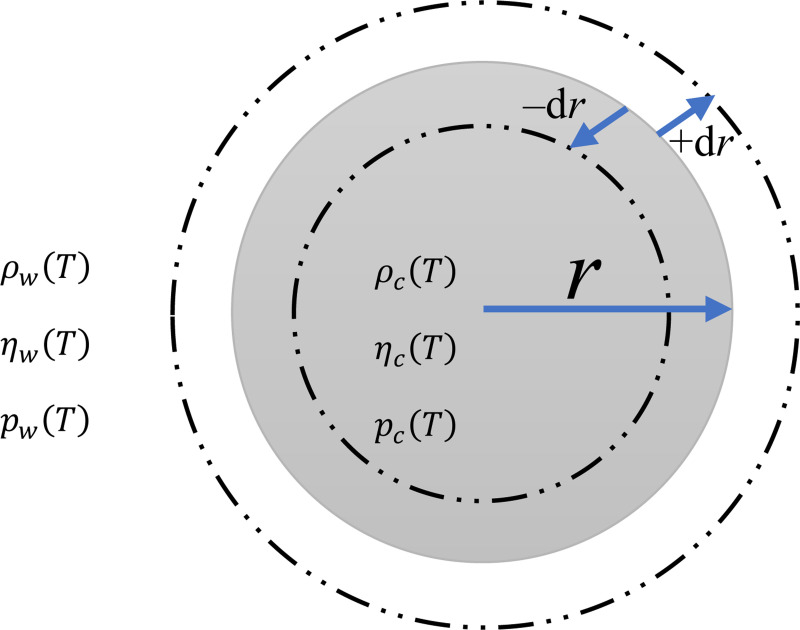
A sketch illustrating the testing of the temperature-size rule using physical first principles.

Here, we propose a physics-based approach to investigating the temperature dependence of phytoplankton size structure. One can model the cell as a confined fluid in contact with an external fluid (the sea/freshwater), where changes in cell size (±dr) are treated as motion relative to a reference point (cell radius –r), with the rate of change (dr/dt) representing the velocity of size variation $\left(v_{r}\right)$ as sketched in Figure 1.

This change—whether expansion or contraction—can be described using the compressible Navier-Stokes equation, governed by the balance of incoming and outgoing fluxes. The temporal evolution and gradients of temperature-dependent intracellular mass density $\left(\rho_{c}(T)\right)$, viscosity $\left(\eta_{c}(T)\right)$, and pressure $\left(p_{c}(T)\right)$, relative to their extracellular counterparts $\left(\rho_{w}(T),\eta_{w}(T),p_{w}(T)\right)$, determine whether the cell size increases or decreases with temperature rise. The temperature response of intracellular and extracellular viscosity, mass density, and pressure can be measured experimentally under different metabolic states and nutrient availabilities.

Viscosity fluctuations can impact swimming/sinking cost, based on the Stokes law, and the prey-predator interactions [31]. When motility speeds surpass a certain threshold, they induce deformations in the fluid flow, which act as signals to predators [20]. Higher viscosities can dampen these signals, reducing encounter rates in predator-prey interactions and thus influencing prey capture and mortality rates [28].

Aside from the temperature-dependent aspect of viscosity, it’s important to note that viscosity is not necessarily constant in the ocean. Phytoplankton motility takes place within a viscosity gradient, suggesting that viscosity is influenced not just by water properties but also by the biological activity of microorganisms within shear-induced aggregates or associated with extracellular polymeric substances (EPS) of high molecular weight, as well as light-induced lysis [28].

Recent microrheological techniques [28] have revealed that approximately 45% of the studied phytoplankton species (including cultures of diatoms, dinoflagellates, and haptophytes) experience viscosity levels up to 40 times that of artificial seawater within a 30 μm radius from the cell (phycosphere). According to these authors [28], the relative viscosity is, on average, 2.6 times higher near the cell wall due to the production of extracellular polymeric substances, up to 30% higher near lysis and dead cells, and more than one order of magnitude higher within aggregates. How do these viscosity gradients affect the motility of phytoplankton, and do they employ any strategies in response? Microfluidic experiments involving *Chlamydomonas reinhardtii* by Stehnach *et al.* [38] indicate that these organisms encounter high local viscosities, which decrease their speed. Nevertheless, they exhibit adaptability by changing their strategy and reorienting down the gradient [38].

Recent studies [20,21,258–260] consistently suggest that swimming speed of unicellular eukaryotes shows either no dependency on cell volume or a weak sub-linear dependence. However, some older references (e.g. [201]) propose that intermediate-sized dinoflagellates display the highest swimming speeds. A challenge encountered in the analyzed data across the literature is the limited sample size, which often fails to encompass the entire spectrum of volume and speed ranges. Additionally, data collected from different studies covering a wide array of sizes and speeds may vary in terms of temperature, physiological conditions, and other influencing parameters. Hence, the initial approach to addressing this issue involves acquiring a comprehensive and uniform dataset that spans across all size and speed ranges. A similar situation applies to the sinking speed of species. Generally, it is reported that the sinking rate increases linearly with cell size [77,261], however, this relationship is not considered universal due to the high uncertainties between the scattered empirical data and the theoretical models.

**Figure 2:**
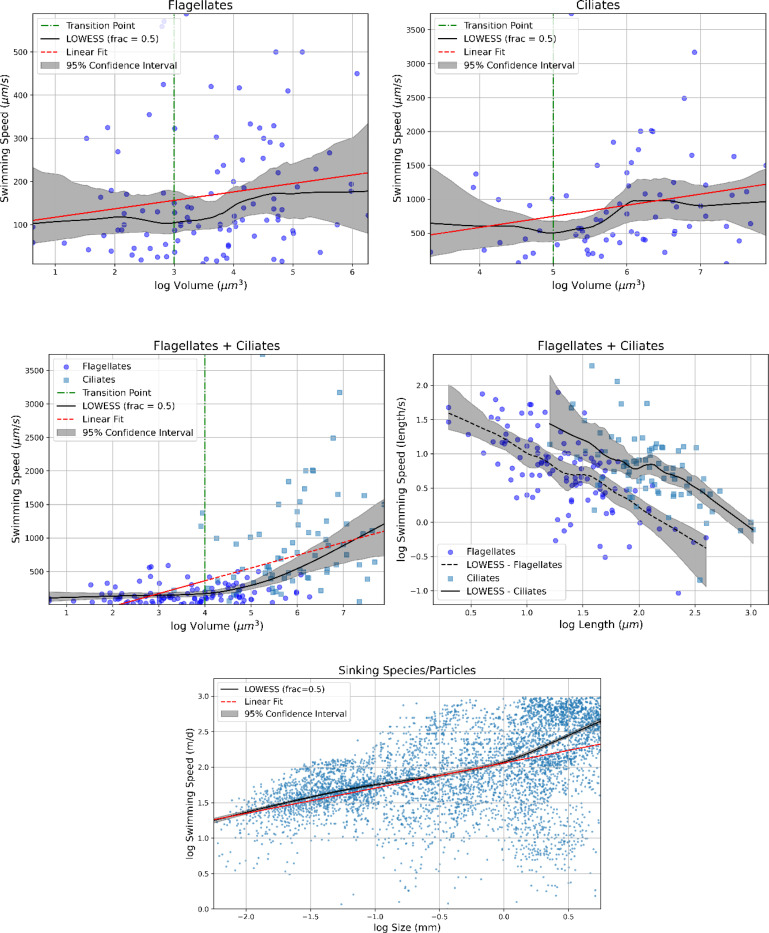
LOWESS regression curves depicting the semi-log volume[μm^3^]-speed[μm/s] relationship for flagellates (top-left), ciliates (top-right), a combination of flagellates and ciliates (middle-left), log-log length[μm]-speed[length/s] relationship for both flagellates and ciliates (middle-right), and the log-log size[mm]-speed[m/d] plot of sinking species/particles (bottom).

One strategy for dealing with highly scattered data involves identifying nonlinear patterns through smoothing techniques. One such method is Locally Weighted Scatterplot Smoothing (LOWESS) [262], which segments the dataset into subregions, conducts linear regressions within each subregion, and then connects them using a cubic function. Here, we apply this approach to analyze the swimming speed versus size data of 111 flagellates and 78 ciliates collected by Lisicki *et al.* [259] from different references, along with data on sinking speed of living species and other marine particles versus size obtained from 5650 sinkers collected in [261]. The data on flagellates and ciliates follow a log-normal distribution, whereas the data on sinking species/particles exhibit positive skewness, resembling a gamma distribution. Additionally, the log-transformed data of these sinking species/particles display a bimodal distribution with negative skewness. Prior to conducting the analysis, we initially eliminated outliers from the data utilizing the z-score approach, employing the standard deviation and mean. We filtered the data for z-scores exceeding 2. This process resulted in the removal of 8 data points pertaining to flagellates, 7 data points corresponding to ciliates, and 578 data points related to sinking species/particles. The result is shown in Figure 2.

When should these nonlinearities be considered in data analysis? Nonlinear data analysis is necessary when nonlinear regression is statistically significant with a considerable confidence interval and when the observed nonlinearity, along with its associated variations and noise, has a meaningful physiological interpretation. In Figure 2, none of the nonlinearities are significant except in the middle-left panel, which shows a transition in speed across species from flagellates to ciliates. In Figure 2, the semi-log plots in the top row—flagellates (upper left) and ciliates (upper right)—do not demonstrate a statistically significant nonlinear or linear pattern. However, the steepest slopes of the LOWESS curves in flagellates and ciliates respectively are > 3 and ~ 4 times greater than the slopes of the corresponding linear regressions. Combining data for flagellates and ciliates (middle-left) reveals a statistically significant increase in swimming speed (in μm/s) for log volumes (in μm^3^) greater than 4 (vertical green line), attributable to taxonomic differences. This suggests an average increase in speed during the transition from flagellates to ciliates. The middle-right figure represents the log-log plot of swimming speed (in body lengths per second) versus body length (in μm) for both flagellates and ciliates. As shown, swimming speed relative to body length decreases with increasing cell size in both groups, exhibiting similar slopes. However, this analysis represents speed-to-length ratio versus length (a spurious correlation of ratios), meaning that since both axes include length, it may introduce a statistical artifact rather than capturing a true biological trend. Given the lack of clear linear or nonlinear patterns in the top-left and top-right figures, along with substantial noise in the data, the observed decreasing trend and similar slopes between flagellates and ciliates in the middle-right figure could be an artifact of division bias. Therefore, this trend requires further investigation in future studies. In sinking species/particles (bottom, log-log plot), the LOWESS curve diverges from linear regression for log sizes (in mm) exceeding zero with a high confidence interval. Based on this analysis, we cannot yet confirm or refute the hypothesis of a relationship between size and speed in phytoplankton swimmers and sinkers. We also analyzed the dataset compiled by Velho *et al.* [263], which includes measurements of body length and swimming speed for bacteria, archaea, flagellates, ciliates, and spermatozoa. Our findings align with those discussed in Figure 2. The only distinct pattern is observed in spermatozoa, which show a slight negative correlation between swimming speed and body length, suggesting that larger sperm tend to swim more slowly. However, this correlation is weak.

## Conclusion

Sinking diatoms tend to thrive in turbulent waters where vertical mixing occurs [264,265], while dinoflagellates are more common in regions with established stratification [266]. Sinking diatoms quickly initiate a surface bloom when nutrients are brought up through vertical mixing, followed by a subsurface bloom near the thermocline due to an influx of reactive nitrogen [15]. On the other hand, dinoflagellates use their swimming ability to counteract their physiological disadvantages (having lower photosynthesis rates, due to low chlorophyll to carbon ratio [8], and higher metabolic costs compared to diatoms), allowing them to coexist with sinking diatoms [15].

We are still some distance away from achieving a comprehensive cost-benefit analysis of phytoplankton swimming and sinking behaviors, with numerous uncertainties remaining. Some less-explored topics encompass the cost-benefit analysis of dynamic strategies employed in response to migratory or turbulent conditions, such as sudden bursts of movement, alterations in shape and orientation, fluctuations in mass density, formation of chains, subdivision into subpopulations, interactive efforts between hosts and epibionts, viral infections, bioluminescence, depth-dependent environmental and physiological changes, temperature dependent motility variations, and viscosity gradients.

In this review, we synthesized diverse aspects of the costs and benefits of phytoplankton motility, ranging from the level of a single cell to the scale of the entire ocean. Our goal was to achieve an integrative understanding of this phenomenon by utilizing the current knowledge available in the literature and proposing new avenues for future investigations. We conducted a thorough examination of the motility costs of a model dinoflagellate, estimating these costs relative to the organism’s available energy resources.

We find that the efficiency and construction costs associated with sinking/rising-related machinery, as well as the swimming mechanisms of surface-distorted cyanobacteria, remain largely unexplored. Phytoplankton sinkers/risers are active, motile microorganisms with invisible buoyancy regulation mechanisms. We investigated the construction costs and potential sources of internal energy dissipation related to motility in diatoms, cyanobacteria, and coccolithophores. This can aid biophysicists in developing equations to model the internal efficiency of buoyancy-regulating phytoplankton. We propose that the rapid bursts of movement followed by near-zero speeds in diatoms could be modeled similarly to the inflation and deflation of a balloon or a jumping-like motion with a stochastic nature. Additionally, we identified and estimated the construction cost of T4P machines in *Synechocystis*, addressing a gap in the literature.

The physics underlying the shape-related external efficiency of sinkers remains an open problem. Specifically, why do spherical sinkers achieve higher speeds compared to sinkers with other geometric shapes, while it is understood that prolate-shaped swimmers exhibit higher speeds than swimmers with spherical or oblate shapes?

Oblate-shaped phytoplankton swimmers with an aspect ratio of ~0.01 exhibit remarkably high swimming external energy dissipation, up to 10^6^ times that of prolate-shaped microswimmers with an aspect ratio close to 100, making swimming a highly inefficient strategy. Why do these species possess swimming abilities despite such inefficiencies? For example, in bacteria, flagella serve multiple functions beyond swimming, including mechanosensing [267], wetness sensing [268], adhesion [269], and contributing to virulence [269].

The temperature dependence of cell size, as we proposed, can be examined through fundamental physics principles, considering the balance between cytoplasmic and environmental viscosity, mass density, and pressure in response to temperature changes under varying nutrient availability. This approach will identify the conditions under which cell size increases or decreases with temperature.

It is still early to conclude whether there is a clear relationship between motility speed and the size of plankton species. This remains a gap in the literature, pending the achievement of coherent datasets that cover the full range of microorganism sizes and speeds, all in stable physiological and environmental conditions. Such datasets may need to be collected on a phylum-by-phylum, genus-by-genus, or species-by-species basis.

## Figures and Tables

**Table 1:** The flow velocity equations modeled for various phyla of flagellated phytoplankton [20,47], are generated from time-averaged point forces with magnitude F applied at a source point. These equations describe the flow velocity at a field point (x,y,z) in Cartesian coordinates, where $d=\sqrt{x^{2}+y^{2}+z^{2}}$ represents the distance between the origin and the field point. The chrysophyte studied in [20] is a sessile cell that generates a feeding current using a single beating flagellum (see Movie S2 in [20]). This beating flagellum can be modeled as a point force acting at the origin, directed along the z-axis in 3D space, with x, y, and z representing the spatial coordinates of the beating flagellum. The resulting velocity field resembles that of a stokeslet, which is a fundamental solution to the Stokes equation in free space (without boundaries) [52]. In the velocity field equation, η represents the viscosity of the fluid. The raphidophyte studied in [20] propels itself by generating force at the front of its body through the motion of its flagella (see Movie S1 in [20]). In these organisms, the flagellar beat is typically oriented so that the flagellum pulls the organism forward through the surrounding fluid, rather than pushing it from behind (meaning the force applied by the flagellum is opposite to the force applied by the cell body). For the derivation of the equation, refer to the supporting information in [49]. The haptophyte and chlorophyte studied in [20] are assumed to propel themselves using a three-stokeslet quadrupole point force model, where two or more flagella generate, mainly, lateral propulsive forces of equal magnitude, pushing the flow backward, while the opposing drag force of the moving cell body balances the sum of these propulsive forces (see Movie S4 in [20]). The velocity field equation is developed in [50].

Phylum	Flagellar arrangement	Point force model	Equation	Ref.
Chrysophyte	One beating flagellum	Stokeslet: A single point force	$v=\frac{F}{8\pi \eta }\frac{\sqrt{x^{2}+y^{2}+4z^{2}}}{d^{2}}$	Supplementary information in [20].
Raphidophyte	One beating flagellum + Opposing force of the cell body	Puller stresslet: Two opposing equal point forces at positions (x=0,y=0,z=±b)	$v=\frac{Fb}{4\pi \eta }\frac{\left|x^{2}+y^{2}-2z^{2}\right|}{d^{4}}$	
Haptophyte Chlorophyte	One or more lateral flagella + Opposing force of the cell body	Three-stokeslet quadrupole: Two opposing equal point forces at positions (x=±a,y=0,z=0) and a third, doubled, point force applied at the origin	$v=\frac{Fa^{2}}{8\pi \eta }f(d)$ *	
Mixotrophic Haptophyte		Described in the text	$v=\frac{F}{3\pi \eta r}f(R,\theta )$**	Equation (3) in [47].

* $f(d)=\frac{\sqrt{4x^{4}\left(x^{2}+21z^{2}\right)-3x^{2}\left(y^{2}+z^{2}\right)\left(y^{2}+13z^{2}\right)+{\left(y^{2}+z^{2}\right)}^{2}\left(y^{2}+16z^{2}\right)}}{d^{6}}$

** $f(R,\theta )=1-\frac{3\left(1+\sin^{2}\theta \right)}{4R/r}-\frac{1-3\sin^{2}\theta }{4{\left(R/r\right)}^{3}}$

**Table 2:** Summary of motility costs for various swimmers and buoyancy-regulating organisms, including flagellated phytoplankton, cyanobacteria, diatoms, and coccolithophores, as presented in this paper.

**Phytoplankton Motility Costs**	Swimming Machineries	Construction costs	- Flagella synthesis, remodeling, and degradation (genetically-controlled length fluctuations, and loss rates). - Crystalline surface layer, SwmA glycoproteins, SwmB polypeptides, and peptidoglycan-associated proteins. - Type IV pilus (T4P) machines.	
		Operational costs	External efficiency	- Geometric shape of the cell body. - Length, number, geometry, angle, and arrangement of flagella
			Internal efficiency	- Elastic bending of the axoneme. - Sliding of microtubules. - Internal viscosity. - Hydrodynamic dissipation caused by motor protein movement generating surface waves. - Proton leak. - Retraction process.
	Buoyancy Regulating Machineries	Construction costs	- Synthesis of gas vesicles and organic osmolytes. - Channels mediating water influx/efflux in vacuoles. - Proteins responsible for ion exchange of heavy and light ions in vacuoles. - Frustule formation. - Formation of coccolith layers.	
		Operational costs	External efficiency	- Geometric shape of the cell body.
			Internal efficiency	- Ratio of gas space to protein wall volume in gas vesicles. - Turgor or hydrostatic pressure - Elastic modulus of gas vesicles. - Density change alignment between protoplasm and frustule. - Cell expansion (elastic energy of morphological changes). - Hydrodynamic dissipation. - Solutes leakage. - Jumping behavior. - Continuous calcification under nutrient-limited conditions.
Other Costs (for both swimmers & sinkers)	Direct costs: - Increased speed due to viruses/predator risk, and turbulence. - Environmental high local viscosity gradients. - Navigation costs. - Cost of rotational motion. - Reorientation and change in motion direction requiring dynamic body shape adjustments, mass density modifications, and alterations in center of mass and buoyancy, which impact both external and internal motility efficiency. - Chaotic path. Indirect costs: - Defense mechanisms, along with nutrient and light limitations during migration, reduce total metabolic power, leading to relatively higher motility costs.			
